# Network-based characterization of latent co-testing patterns in antimicrobial susceptibility testing

**DOI:** 10.1016/j.isci.2026.117421

**Published:** 2026-09-22

**Authors:** Ebenezer Awotoro, Aleksandar Anžel, Chisom Anyabolu, Niklas Willrich, Georges Hattab

**Affiliations:** 1 Center for Artificial Intelligence in Public Health Research (ZKI-PH), Robert Koch Institute, 13353 Berlin, Germany; 2 Department of Mathematics and Computer Science, Freie Universität Berlin, 14195 Berlin, Germany; 3 Department of Infectious Disease Epidemiology, Robert Koch Institute, Berlin, Germany

**Keywords:** antimicrobial resistance, diagnostic stewardship, shadow antibiograms, network analysis, co-testing patterns, similarity metrics, community detection and antimicrobial susceptibility testing

## Abstract

Antimicrobial resistance (AMR) surveillance depends on antimicrobial susceptibility testing, yet antibiotic panels vary by pathogen cohort, specimen, year, ward, and care setting. We term these empirically reconstructed testing architectures shadow antibiograms: structures that shape which susceptibility results enter surveillance datasets and which resistance relationships can be observed. We analyzed approximately 13 million bacterial isolates from Germany’s National Antibiotic Resistance Surveillance system (2019–2023) across six WHO BPPL-aligned operational cohorts. Co-testing networks were constructed using Jaccard, Dice, cosine, and phi-coefficient similarity, evaluated with Fisher’s exact tests and Benjamini-Hochberg correction, and partitioned with multi-resolution Louvain community detection. Networks contained three to four dominant co-testing communities; false discovery rate (FDR) retention ranged from 89 to 100% across cohorts and specimen types. Jaccard provided the best balance of coherence, stability, antimicrobial-class alignment, interpretability, and edge retention. This work provides one of the first national-scale empirical characterizations of shadow antibiogram structure, supporting diagnostic stewardship and bias-aware AMR surveillance interpretation.

## Highlights

• Shadow Antibiograms reveal latent antibiotic co-testing architecture in AST data
• Network analysis of ∼13M German ARS isolates across six WHO BPPL pathogen cohorts
• Urine co-testing networks are stable; blood culture networks vary by context and year
• Supports diagnostic stewardship and bias-aware interpretation of AMR surveillance

## Graphical abstract

**Figure:**
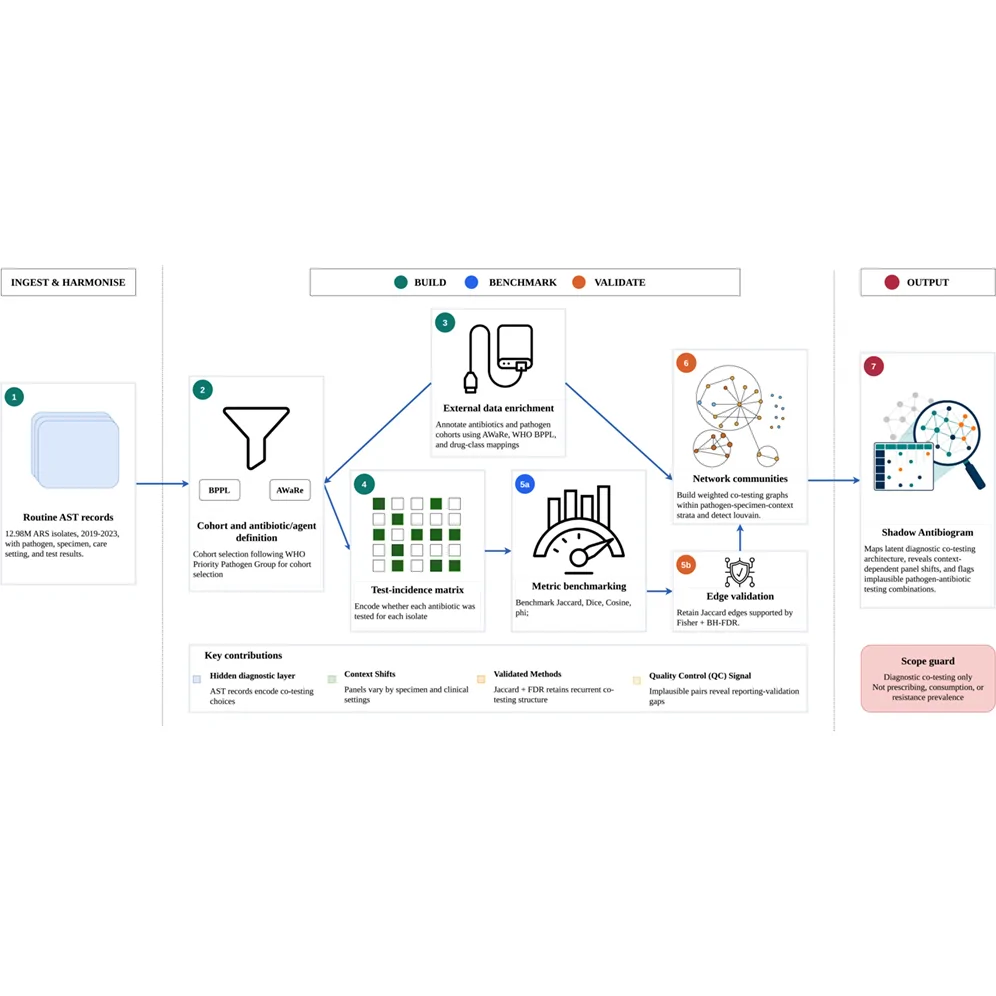

## Teaser

Public health; Gene network; Clinical microbiology

## Introduction

Antimicrobial resistance (AMR) is a major global health threat,^1^ causing an estimated 1.14 million directly attributable deaths among 4.71 million associated deaths in 2021.^2^^,^^3^ Projections suggest that economic losses could total US $1 trillion by 2050.^4^ High-income settings are profoundly affected: in 2019, for example, in Germany, there were an estimated 9,600 attributable and 45,700 associated deaths, largely driven by *Escherichia coli*, *Staphylococcus aureus*, *Klebsiella pneumoniae*, *Enterococcus faecium*, and *Pseudomonas aeruginosa*.^5^ International surveillance networks like GLASS and EARS-Net provide harmonized reporting frameworks,^6^^,^^7^ complemented by WHO AWaRe and Bacterial Priority Pathogen List (BPPL) prioritization lists.^8^^,^^9^ However, antibiotic susceptibility testing (AST) remains highly variable. Local laboratory workflows and stewardship policies influence which antibiotics are tested and reported, meaning that surveillance data reflect both microbial epidemiology and the underlying diagnostic context.^10^^,^^11^^,^^12^^,^^13^

Variation in testing practices directly impacts the interpretation of resistance rates.^10^^,^^12^^,^^14^ Selective and cascade reporting, which is common in some parts of Europe and the US, can withhold or suppress up to 20% of AST results, thereby influencing both clinical prescribing and the composition of surveillance datasets.^12^^,^^15^ In Germany, although cascade reporting is not formally documented, similar mechanisms are likely to influence reported patterns.^16^^,^^17^ This raises a fundamental question: which resistance patterns remain observable given existing testing behaviors?^18^^,^^19^

Two structural factors drive diagnostic variation. First, selection process bias stems from institution-specific panels that vary by clinical setting (e.g., intensive care unit [ICU] vs. outpatient care), making resistance estimates partly dependent on local testing choices.^10^^,^^12^^,^^17^^,^^20^^,^^21^^,^^22^ Second, automation bias could arise when laboratories over-rely on manufacturer defaults specifying diagnostic routines in AST panels, reducing independent verification and constraining observable resistance patterns to preconfigured testing protocols.^23^^,^^24^^,^^25^ This is particularly true where lab governance is limited.

These influences give rise to the concept of the shadow antibiogram (SA): the empirically reconstructed structure of co-testing patterns that determines data availability and observable resistance relationships. While co-testing patterns are raw observations, the SA is the systematized framework representing the implicit structure that emerges from aggregated diagnostic decisions. In practice, identical resistance rates can arise from different clinical contexts, which makes them difficult to compare without knowledge of the testing patterns involved. From a statistical perspective, AST results are rarely missing completely at random (MCAR). Instead, they frequently go missing due to institutional policies and stewardship constraints.^12^^,^^15^^,^^26^ Analytical workflows that ignore these patterns can introduce significant bias into estimates of resistance prevalence and trends.

This paper focuses on characterizing diagnostic testing architecture: the measurable, empirical patterns of which antibiotics are tested together and how these patterns vary across clinical contexts. We do not quantify or correct for bias introduced by selective/cascading testing in this work. As stated above, AST results are often missing not at random (MNAR), driven by testing protocols, clinical priorities, and stewardship policies. This means observed resistance estimates may systematically misrepresent true population prevalence, particularly for antibiotics tested infrequently or selectively in high-risk cases. By making testing architecture explicit and measurable, SA provides an empirical basis for future bias-aware surveillance modeling. The present paper provides the architectural description needed for such future work but does not itself perform quantitative bias adjustment. To explore these diagnostic structures, we apply network analysis to AST co-testing patterns. Within Germany’s Antibiotic Resistance Surveillance (ARS) system, laboratories independently design their testing menus according to local priorities and resources. Rather than treating this heterogeneity as noise, we view it as a potential source of insight into diagnostic behavior, consistent with treating complex biomedical datasets as structured abstractions that benefit from careful visual encoding and analytic design.^27^ Network methods have previously been used to examine cross-resistance patterns.^28^^,^^29^^,^^30^ However, large-scale analyses of co-testing networks remain limited in scope and breadth. Such methods usually identify non-overlapping communities that offer a simplified representation of real-world testing practices, where antibiotics may belong to multiple logical or operational panels. While prior work has conceptualized the idea of diagnostic bias, it has not systematically examined how co-testing patterns vary across facilities or over time.^30^^,^^31^ Understanding such variations could help to contextualize AMR surveillance in practice.

Crucially, the SA does not visualize antibiotic prescribing or consumption patterns. Instead, it reflects diagnostic intentions and the implicit decisions that determine which antibiotic susceptibility results enter the surveillance record in the first place. We address three research questions using records from ARS.

1 What communities of antibiotics emerge from empirical co-testing patterns?
2 How do similarity metrics and network algorithms influence the extraction of robust, interpretable co-testing structures from sparse AST data?
3 How do co-testing networks vary across time and clinical contexts, and what factors contribute to this heterogeneity?

Our work makes the following three main contributions:

1 We explore the SA concept using a network-based approach that characterizes diagnostic co-testing patterns (rather than resistance outcomes alone).
2 We develop and benchmark a computational framework for sparse, high-dimensional AST matrices, integrating multiple similarity metrics, community detection, false discovery rate (FDR) control, and stability assessment.
3 We quantify context-dependent heterogeneity in co-testing networks across clinical settings and specimen types, making variations in diagnostic behavior measurable and observable.

## Results

### Dataset composition and representativeness

The study analyzed 12.98 million bacterial isolates collected from 2019 to 2023 (Figure 1). Each isolate record comprises of contextual metadata and a binary vector of antibiotic testing indicators (Table 1), derived from raw ARS records through a structured preprocessing pipeline (Figure 2). Ten bacterial genera accounted for 95.0% of isolates, dominated by *Escherichia* (36.4%, *n* = 4,728,867), *Staphylococcus* (16.5%, *n* = 2,138,013), and *Klebsiella* (12.2%, *n* = 1,585,973). The remaining seven genera were *Pseudomonas* (7.5%, *n* = 974,462), *Proteus* (7.1%, *n* = 928,415), *Streptococcus* (5.1%, *n* = 658,597), *Enterobacter* (3.8%, *n* = 492,088), *Enterococcus* (3.7%, *n* = 483,146), *Citrobacter* (2.7%, *n* = 348,531), and *Serratia* (1.6%, *n* = 205,655). Specimen types reflected diagnostic practice: urine (46.3%, *n* = 6,009,092), swabs (16.5%, *n* = 2,148,484), wound samples (16.3%, *n* = 2,120,392), respiratory (5.9%, *n* = 766,231), and blood cultures (4.8%, *n* = 626,129). Age distribution concentrated in older adults, with ages 60–84 years comprising 35.1% of isolates (*n* = 4,562,606), and pediatric submissions (0–19 years) representing 8.0% (*n* = 1,041,194). Geographic representation spanned all 16 German federal states, ensuring national-scale diversity of diagnostic infrastructure. Full distributions are presented in Tables S2–S4.

**Figure 1:**
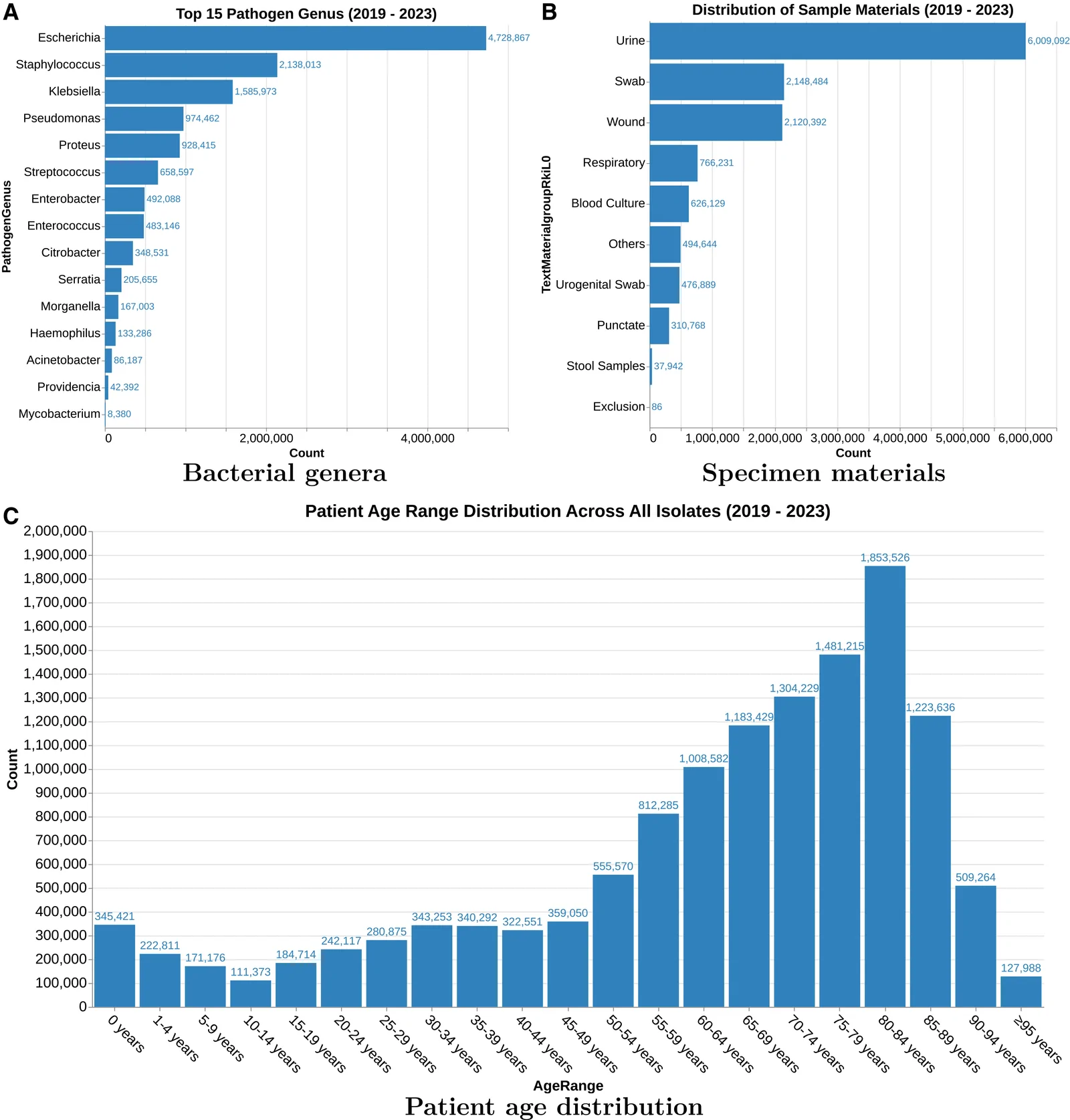
Surveillance dataset overview, 2019–2023 (A) Image A summarizes the broader ARS surveillance dataset at genus level. This descriptive overview is distinct from the downstream network cohort definitions, which followed BPPL-aligned operational pathogen patterns: genus-level Enterobacterales cohorts for *Escherichia*, *Klebsiella*, and *Proteus*; species-level cohorts for *Staphylococcus aureus* and *Pseudomonas aeruginosa*; and a priority *Streptococcus* cohort comprising group A streptococci, *Streptococcus pneumoniae*, and group B streptococci. (B) Specimen material distribution by diagnostic type. (C) Patient age distribution across the study period.

**Table 1:** Structure of the preprocessed dataset

Gram	Pathogen	Genus	Specimen	Care type	Age	Ward	Year	AMC	AMK	AMP	AMS	VAN
Neg	*Escherichia coli*	*Escherichia*	urine	inpatient	70+	ICU	2020	1	1	1	0	1
Pos	*Staphylococcus aureus*	*Staphylococcus*	blood	inpatient	50–59	ward	2021	0	1	0	1	1
Neg	*Klebsiella pneumoniae*	*Klebsiella*	sputum	outpatient	30–39	clinic	2022	1	0	1	0	1
Pos	*Enterococcus faecalis*	*Enterococcus*	wound	inpatient	60–69	internal	2020	0	0	1	1	0
Neg	*Pseudomonas* spp.	*Pseudomonas*	urine	inpatient	70+	ICU	2023	1	1	0	0	1
Pos	*Acinetobacter baumannii*	*Acinetobacter*	blood	inpatient	80+	ICU	2021	1	1	0	1	1
Neg	*Proteus mirabilis*	*Proteus*	urine	outpatient	40–49	clinic	2019	1	0	1	1	0
Pos	*Streptococcus pyogenes*	*Streptococcus*	wound	inpatient	20–29	ward	2022	0	0	1	0	1

Each row corresponds to one clinical isolate and includes contextual metadata and a binary testing pattern indicating whether an antibiotic was tested (1 or 0). Antibiotic codes: AMC, amoxicillin-clavulanate; AMK, amikacin; AMP, ampicillin; AMS, ampicillin-sulbactam; VAN, vancomycin.

**Figure 2:**
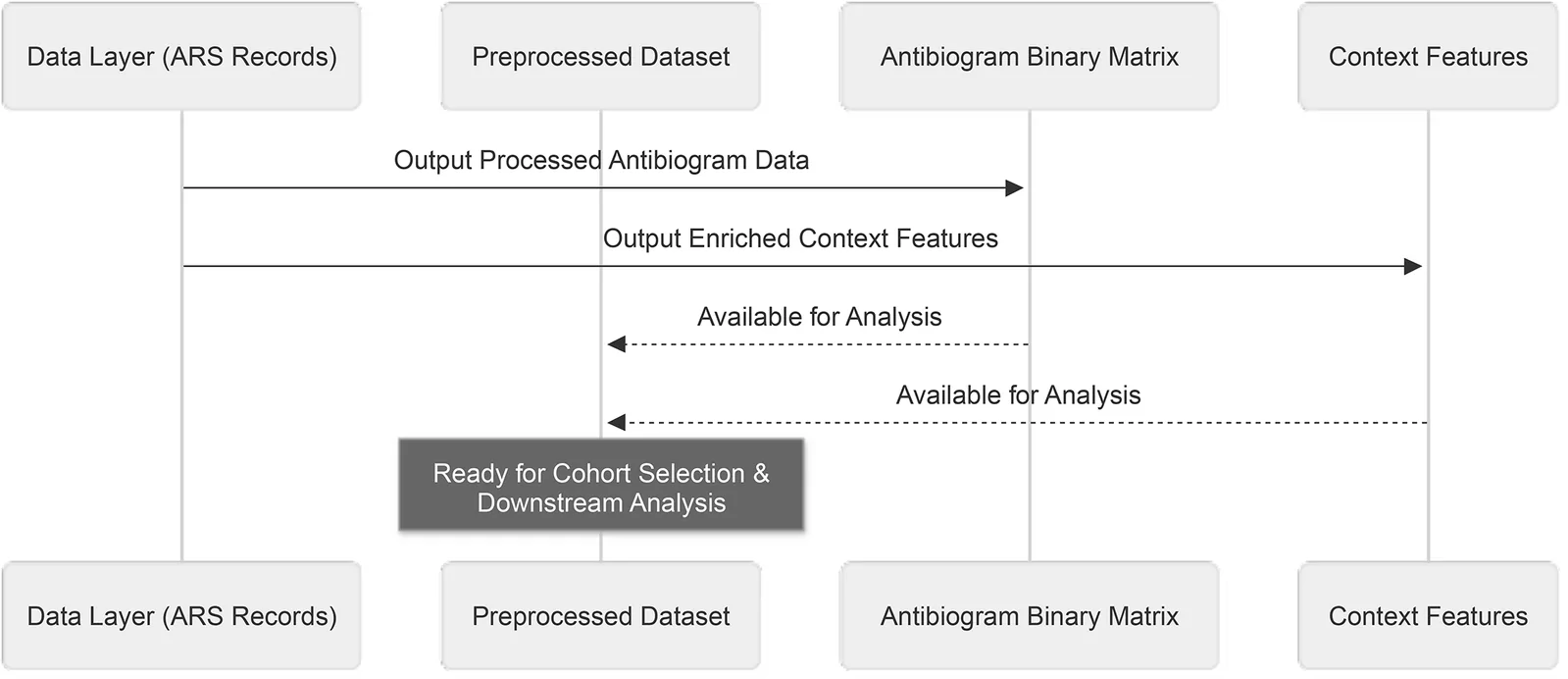
Multi-stage pipeline overview This pipeline uses the ARS record to construct the antibiogram matrix and context features. The main stages are data cleaning, feature filtering and engineering, and selecting the downstream cohort.

### Antibiotic testing trends across WHO AWaRe categories

Testing frequencies stratified by WHO AWaRe classification showed stable but distinct temporal patterns over the study period, as seen in Figure 3. The access group increased from 34.5% of tests in 2019 to 36.9% in 2023, with a transient decline in early 2020 followed by sustained growth. The watch class consistently represented the majority of testing volume, declining slightly in relative contribution from 57.1% to 56.0%, despite increasing absolute test counts. Within the watch group, ciprofloxacin testing increased from 8.4% to 8.9%, while levofloxacin decreased from 7.9% to 5.1%, and moxifloxacin decreased from 3.9% to 1.6%. Reserve-class antibiotics remained stable at approximately 7% of testing volume from 2020 onwards.

**Figure 3:**
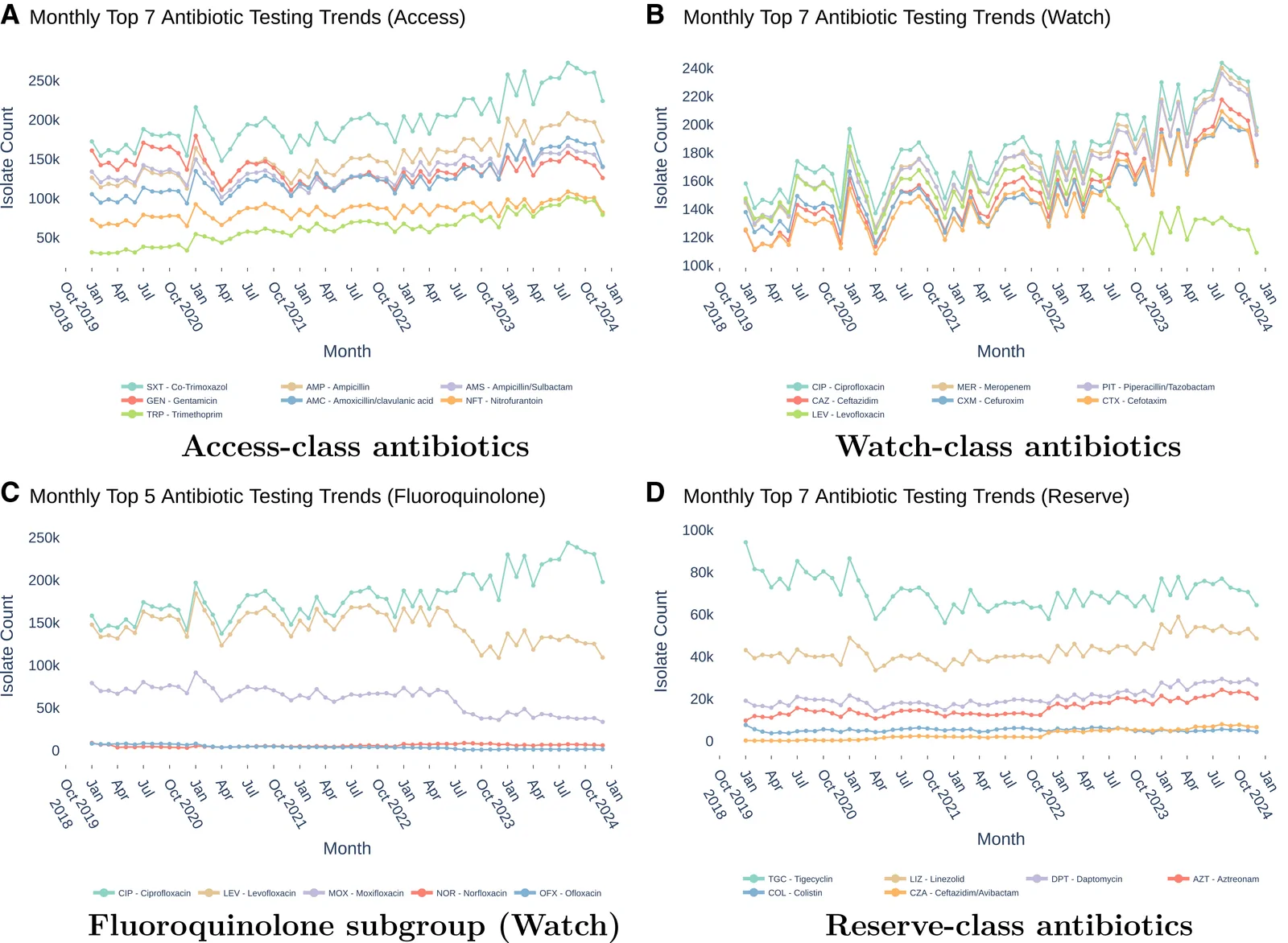
Monthly testing trends across WHO AWaRe antibiotic classes, 2019–2023 (A) Access-class antibiotics increased in relative share, from 34.5% of monthly tests in 2019 to 36.9% in 2023, following a transient decline during early 2020. (B) Watch-class antibiotics consistently dominated testing activity, with mean monthly volumes rising over time but slight proportional decline from 57.1% in 2019 to 56.0% in 2023. (C) Fluoroquinolones (watch subclass) showed divergent trends: modest growth in ciprofloxacin contrasted by sustained declines in levofloxacin and moxifloxacin, consistent with post-2018 regulatory restrictions. (D) Reserve-class antibiotics remained infrequently tested, contributing approximately 7% of monthly tests throughout the study period. Note on interpretation: the AWaRe classification system was developed to inform the prescription and use of antibiotics. In this figure and throughout the manuscript, the AWaRe categories are used solely as a pharmacological classification system for the antibiotics included in the AST panels. Testing volume, defined as the number of susceptibility tests performed, reflects laboratory diagnostic decisions, not the volume of antibiotics prescribed or consumed. These two quantities measure distinct phenomena, and their relationship cannot be inferred from ARS susceptibility data alone. Consumption data (e.g., defined daily doses) are captured by antibiotic stewardship programs and are not recorded in the ARS susceptibility surveillance system.

The AWaRe framework guides antibiotic prescribing and consumption,^8^^,^^32^ but our analyses measure diagnostic testing frequency: which antimicrobial agents laboratories include in AST panels across reporting organizations. Testing frequency reflects laboratory diagnostic choices, including panel composition and cascade reporting, rather than antibiotic consumption. AWaRe-stratified testing volumes should therefore be interpreted as diagnostic testing patterns, not as proxies for antimicrobial use.

### Inter-laboratory heterogeneity in testing coverage

Figure 4 illustrates inter-laboratory variability in antibiotic testing coverage, expressed as the proportion of isolates tested for each antibiotic and stratified by AWaRe classification. Access-group antibiotics exhibited variable median coverage depending on the specific agent: while some access drugs showed relatively high coverage across most laboratories, others had low-to-moderate median coverage with substantial inter-laboratory dispersion. Watch-class antibiotics demonstrated the greatest heterogeneity in testing practices, spanning agents with consistently high coverage across laboratories as well as drugs with lower median coverage that were tested frequently only in a subset of laboratories, as reflected by wide interquartile ranges and occasional high-coverage outliers. Reserve-class antibiotics exhibited the lowest overall coverage, with most agents clustered near zero across laboratories; however, a small number of reserve antibiotics showed comparatively higher and more variable coverage, suggesting selective testing practices in certain laboratories rather than routine inclusion in AST panels.

**Figure 4:**
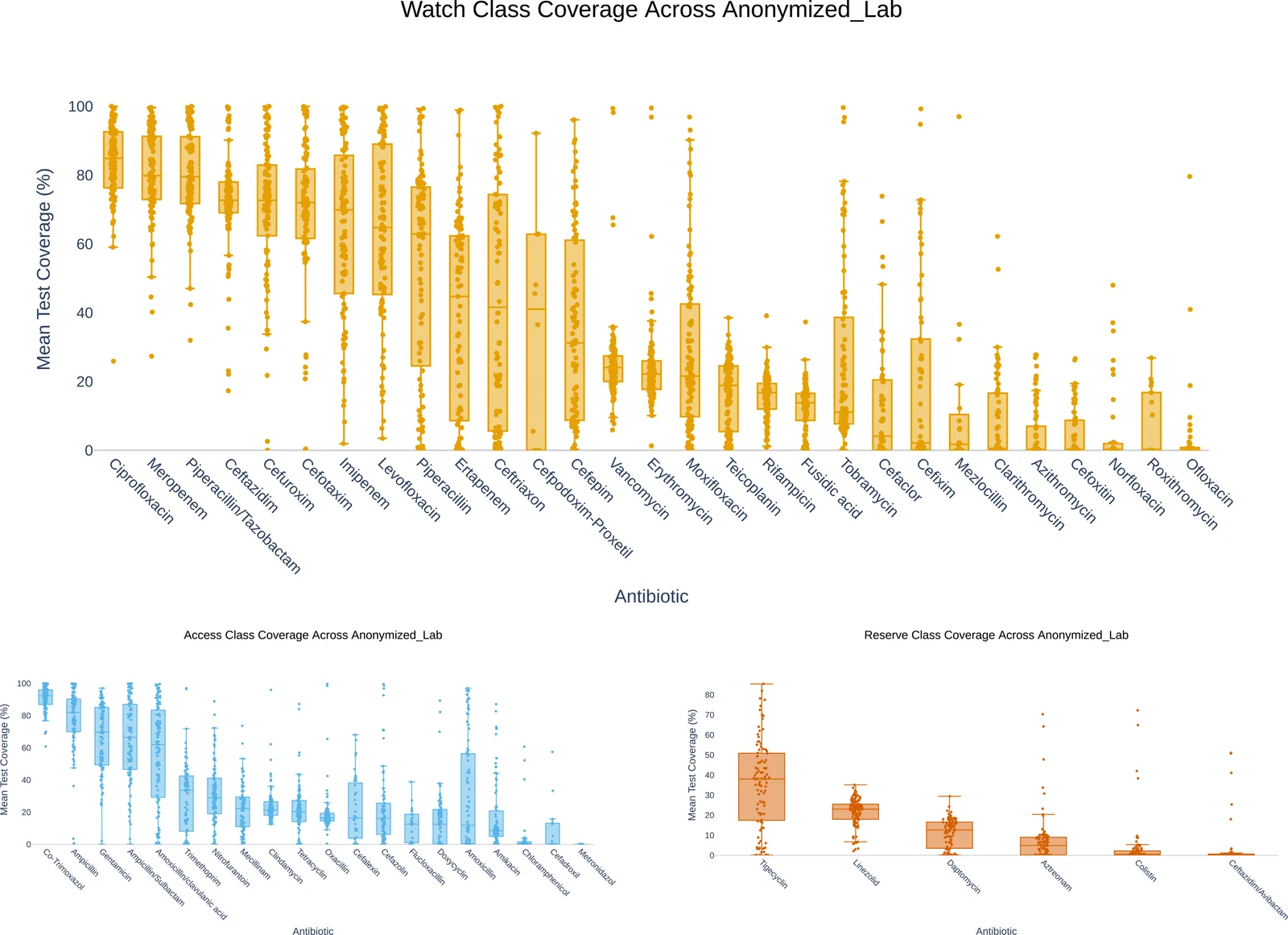
Inter-laboratory distribution of antibiotic testing coverage by AWaRe group Boxplots show mean test coverage (percentage of isolates tested per antibiotic) across laboratories by AWaRe classification. Watch-group antibiotics display wide variability in coverage and dispersion. Access-group antibiotics show variable coverage across agents with differing inter-laboratory spread. Reserve-group antibiotics exhibit low coverage with clustering near zero, alongside occasional high-coverage observations. Boxes represent the interquartile range, lines indicate the median, whiskers denote the whisker range, and points represent individual laboratory values beyond the whiskers.

### Similarity metric evaluation and selection

To reconstruct SAs, a similarity metric must be selected that captures meaningful co-testing relationships while ensuring interpretability and reproducibility across diverse pathogen cohorts and specimen types. We evaluated four candidate metrics: Jaccard, Dice, cosine, and *ϕ*, using a multi-stage validation framework to identify which best preserves clinically meaningful co-testing patterns across pathogen cohorts and specimen types. The following subsections present quantitative performance comparisons from stage 1 (as mentioned in section), statistical robustness testing from stage 2 (as mentioned in section), stability analyses under parameter variation, external alignment with established antibiotic classifications, and visual demonstration of metric influence on community structure.

#### Stage 1: Network quality-based ranking

The phi coefficient achieved the highest intrinsic coherence scores (mean ICS = 6.27) across all six BPPL-aligned operational pathogen cohorts and both clinical materials (blood culture and urine). However, clusterings based on the phi coefficient showed increased network fragmentation compared with the other metrics, dividing AST panels into more selective sub-communities (mean = 18.16 communities).

Jaccard similarity produced high structural coherence (mean ICS = 4.11) and community partitions (mean = 20.38 communities). The cosine and Dice similarity measures showed lower coherence across pathogen cohorts and materials, with a mean ICS of 2.39 and 3.08, respectively. Clustering showed broader and less clearly defined community boundaries. Stability analyses showed that Jaccard and *ϕ* produced reproducible community structures across bootstrap resampling and parameter sweeps (mean Adjusted Rand Index (ARI) of 0.85 and 0.88, respectively), whereas cosine similarity displayed lower reproducibility (mean ARI = 0.78). External validation favored Jaccard, achieving the highest mean hierarchical congruence (0.45), with *ϕ* slightly lower (0.43). Figure 5 illustrates these patterns for *Escherichia* urine isolates at optimized thresholds (*τ*∗ = 0.30, *γ*∗ = 1.0) for each metric. *ϕ* yields tightly constrained subcommunities reflecting highly specific testing combinations. Cosine and Dice generate broader groupings driven by shared testing prevalence. Jaccard provided the most consistent balance between coherence, biological alignment, stability, and clinical interpretability and was selected as the principal similarity measure for all subsequent SA analyses. Detailed performance metrics are reported in Table S12 and in the dataset descriptor.^33^

**Figure 5:**
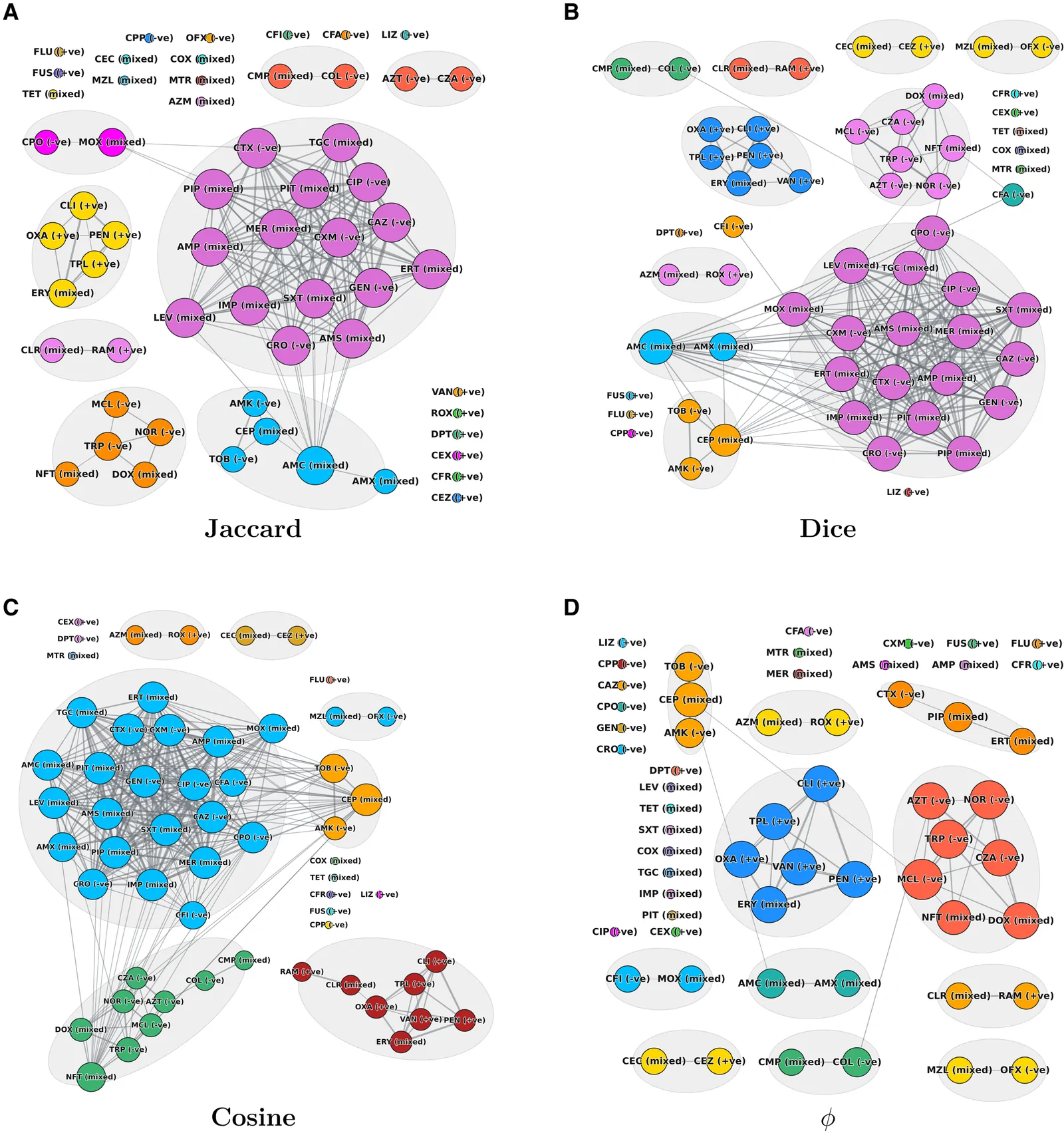
Co-testing network structures across similarity metrics for *Escherichia* blood cultures Visual encoding: Node size is proportional to antibiotic degree (number of statistically supported co-testing connections after FDR correction). Edge thickness reflects the subplot-specific similarity metric shown in each subplot: Jaccard in (A), Dice in (B), cosine in (C), and phi coefficient in (D); heavier edges indicate stronger pairwise co-testing association under that metric. Layout note: node position in the force-directed layout reflects Louvain community assignment and graph aesthetics, not pairwise co-testing strength; spatial proximity between nodes should be interpreted only as a layout aid and must not be read as indicating high joint testing frequency. Color note: node colors represent Louvain algorithmic communities identified independently for each similarity metric. They do not correspond to the WHO AWaRe classification scheme (access = blue, watch = orange, and reserve = vermilion) used in Figures 6, 7, 8, 9, and 10. Networks were generated using optimal parameters (*τ*∗, *γ*∗) identified in stage 1, with threshold *τ*∗, and colored by Louvain community detection. Oval envelopes delineate community boundaries. Metrics exhibit distinct structural behaviors: (A) Jaccard yields compact, clinically coherent communities aligned with routine Gram-negative testing workflows; (B) Dice produces more diffuse communities with weaker modular definition; (C) cosine generates similarly loose structures with limited panel cohesion; (D) *ϕ* forms tighter, more constrained sub-communities due to strong penalization of joint absences. These qualitative differences mirror quantitative findings from stage 1 and justify Jaccard selection for downstream analysis. For interactive exploration, see section A (similarity metrics) in Table S15.

**Figure 6:**
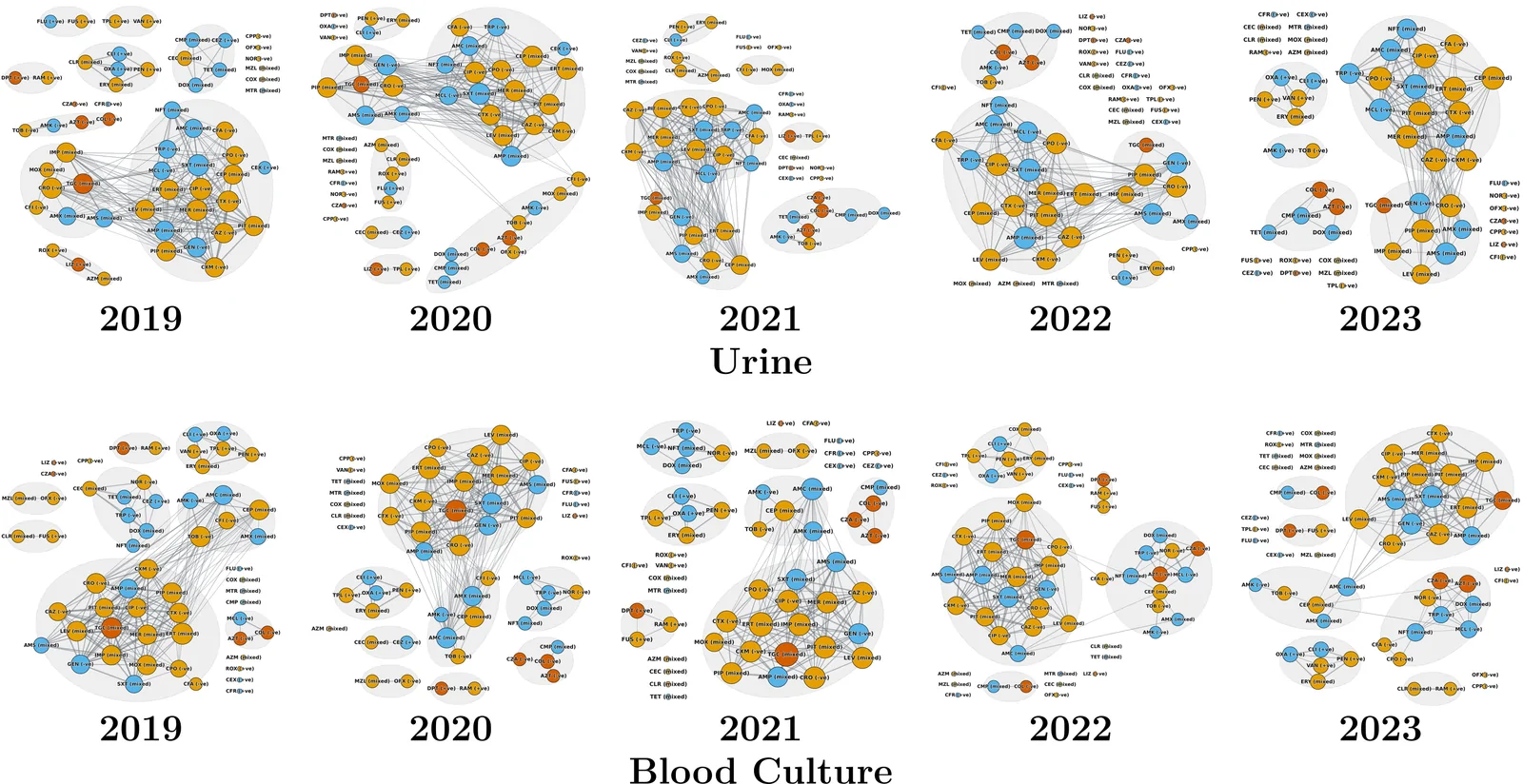
Temporal evolution of co-testing networks for *Escherichia* isolates, 2019–2023 Annual Jaccard-based similarity networks for *Escherichia* derived from urine (top row) and blood culture (bottom row) specimens. Networks were filtered using Fisher’s exact test with Benjamini-Hochberg correction (*q* < 0.05), partitioned using Louvain community detection (*γ* = 1.0), and visualized with a Jaccard similarity threshold (*τ* = 0.3). Node color denotes WHO AWaRe antibiotic category: access (blue), watch (orange), and reserve (vermilion). Node size is proportional to degree, representing the number of co-tested antibiotic connections. Edge thickness corresponds to Jaccard similarity strength, and dashed oval envelopes delineate Louvain communities. Layout note: node position in the force-directed layout reflects Louvain community assignment, not pairwise co-testing strength; spatial proximity between nodes should be interpreted only as a layout aid. Over time, urine-derived networks exhibit increasing edge density and connectivity, whereas blood culture networks remain comparatively sparse and modular across years. For interactive exploration, see section B (temporal trends) and Table S15.

**Figure 7:**
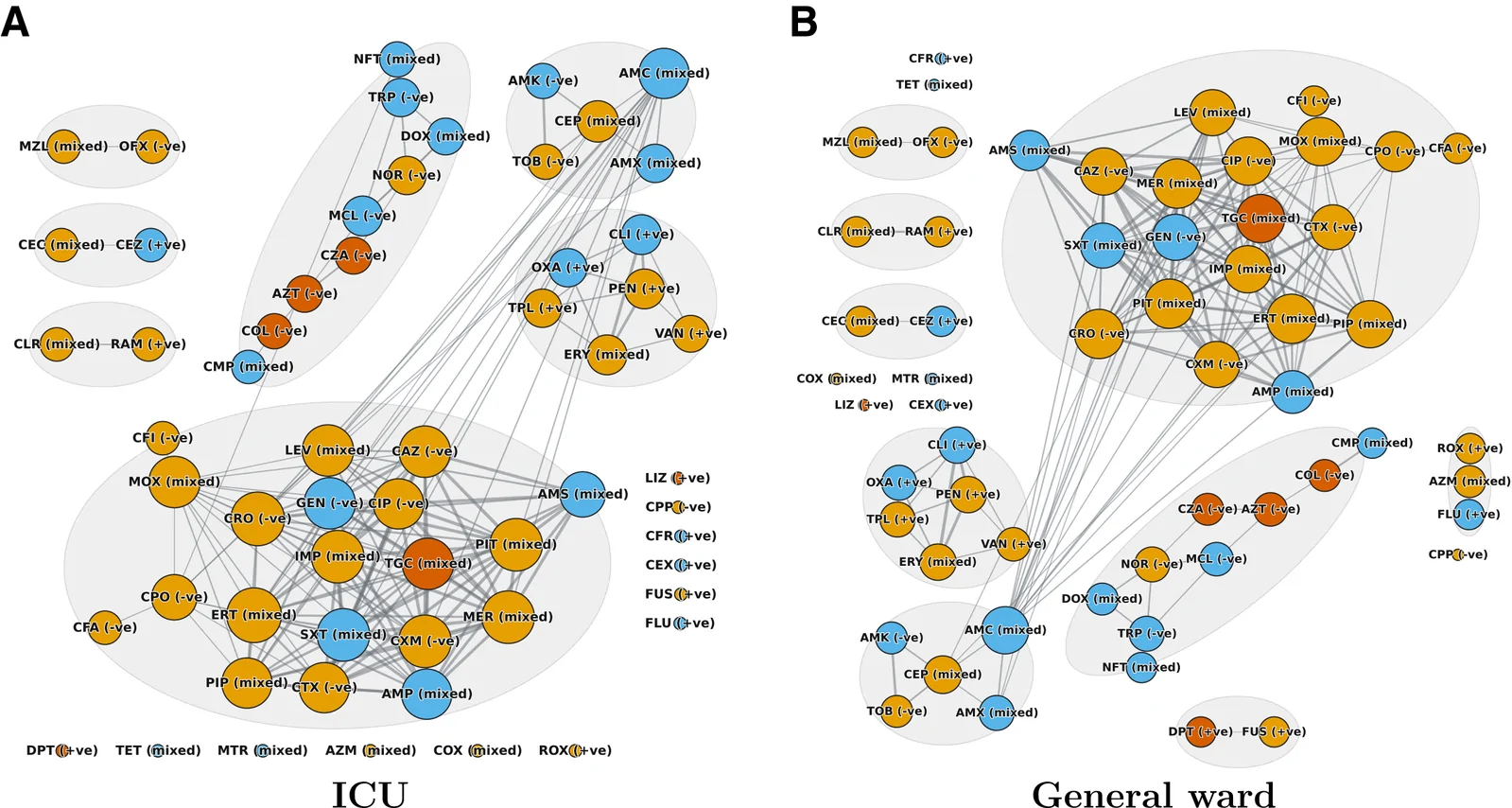
*Escherichia* blood culture isolate co-testing networks stratified by inpatient setting (ICU vs. general ward) Node color indicates WHO AWaRe class (blue, access; orange, watch; vermilion, reserve) and node size reflects testing prevalence. Edges represent Jaccard co-testing similarity (retained if *q* < 0.05), with thickness proportional to similarity strength; dashed envelopes denote Louvain communities. Layout note: node position in the force-directed layout reflects Louvain community assignment, not pairwise co-testing strength; spatial proximity between nodes should be interpreted only as a layout aid. ICU networks (A) show tighter integration and increased inter-module connectivity, whereas general ward networks (B) exhibit more modular structure with clearer peripheral segregation. For interactive exploration, see Table S15, row 18 and row 17.

**Figure 8:**
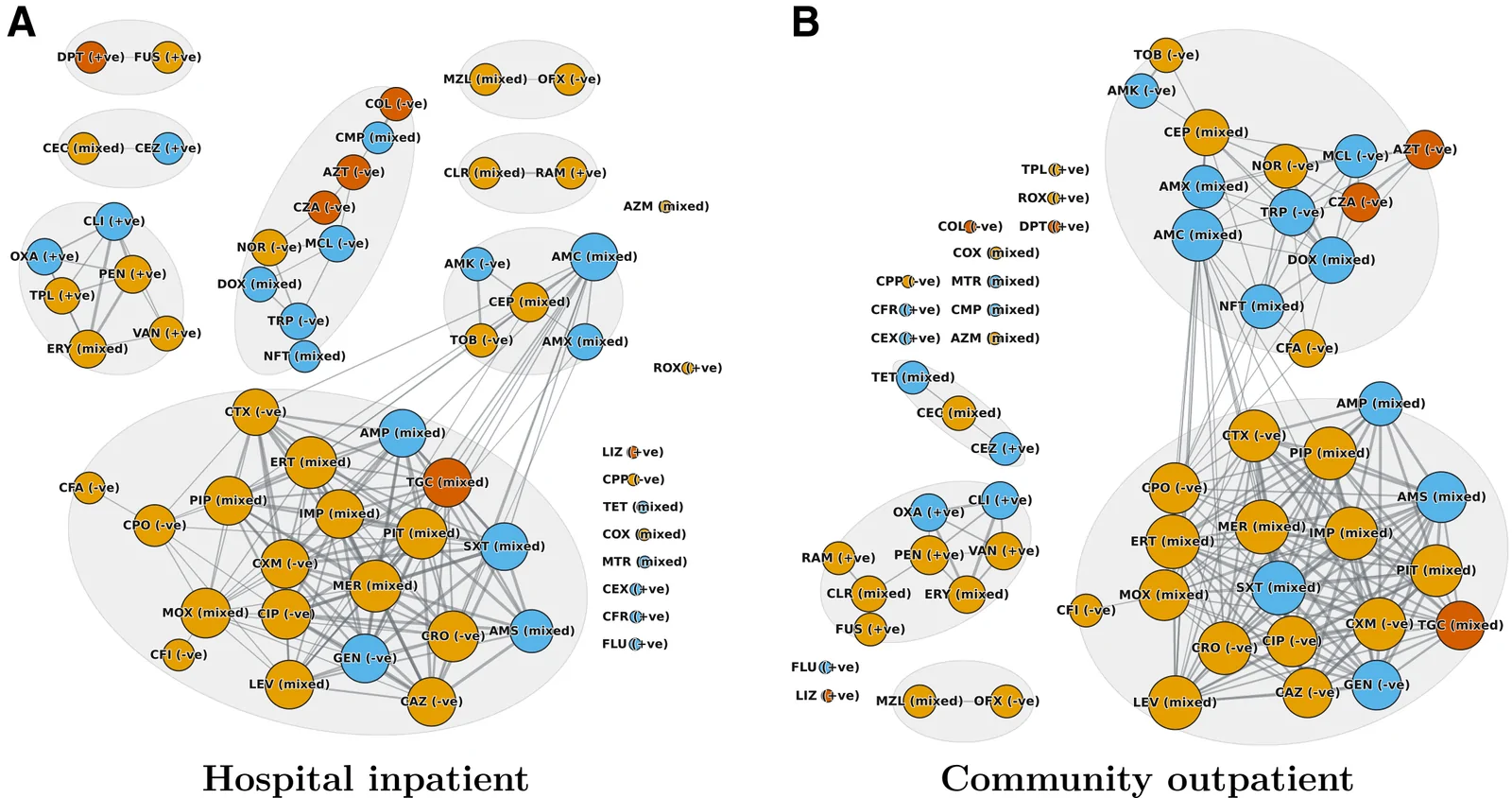
*Escherichia* blood isolate co-testing networks stratified by care setting (hospital inpatient vs. community outpatient) Node color denotes WHO AWaRe class (blue, access; orange, watch; vermilion, reserve) and node size indicates testing prevalence. Edges represent Jaccard co-testing similarity (retained if *q* < 0.05), with thickness proportional to similarity strength; dashed envelopes denote Louvain communities. Layout note: node position in the force-directed layout reflects Louvain community assignment, not pairwise co-testing strength; spatial proximity between nodes should be interpreted only as a layout aid. Images (A) and (B) visualize setting-specific differences in the composition and connectivity of empirical co-testing communities. For interactive exploration, see Table S15, row 15 and row 16.

**Figure 9:**
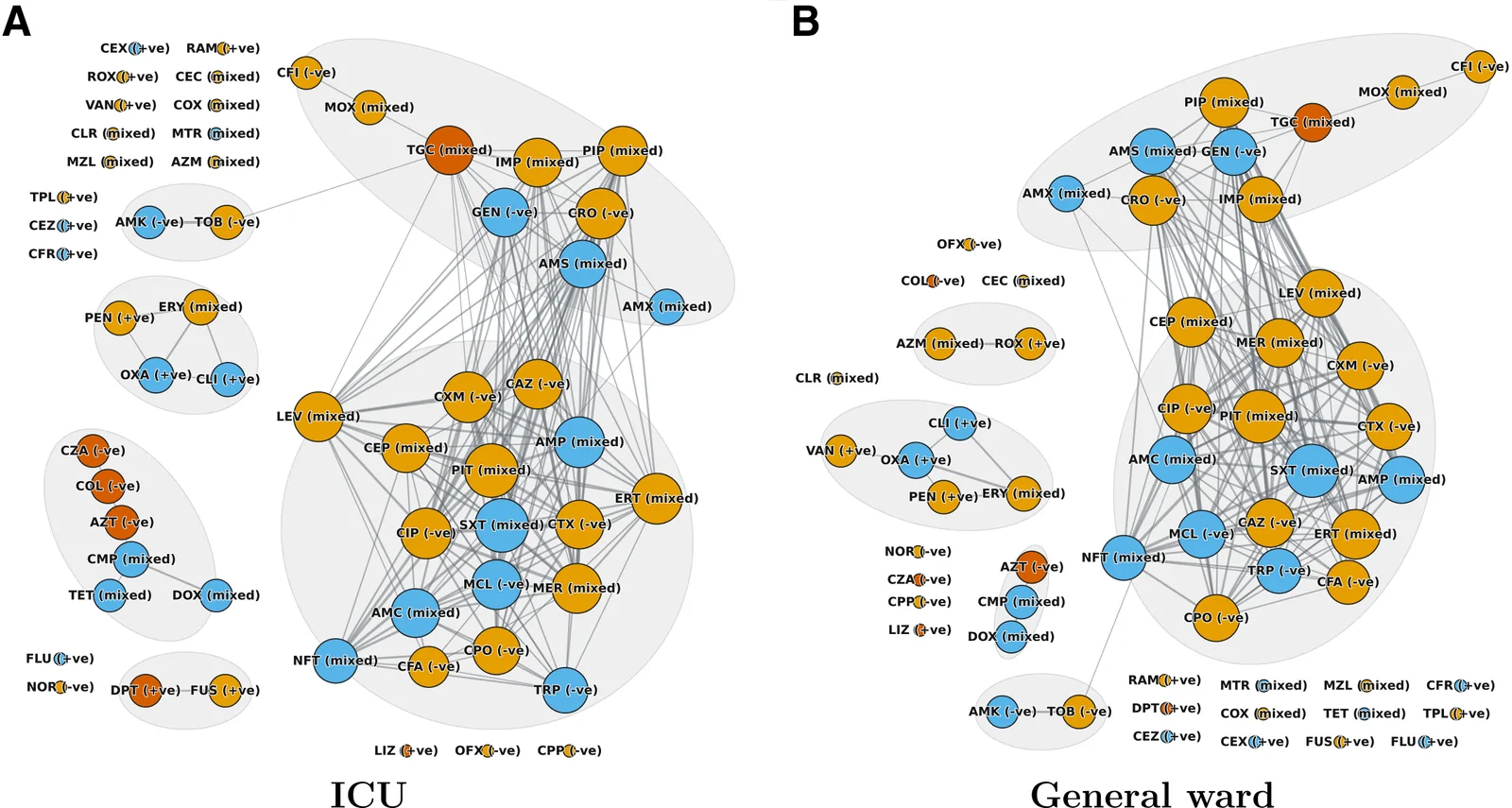
*Escherichia* urinary isolate co-testing networks stratified by inpatient unit (ICU vs. general ward) Node color denotes WHO AWaRe class (blue, access; orange, watch; vermilion, reserve) and node size reflects testing prevalence. Edges represent Jaccard co-testing similarity (retained if *q* < 0.05), with thickness proportional to similarity strength; dashed envelopes denote Louvain communities. Layout note: node position in the force-directed layout reflects Louvain community assignment, not pairwise co-testing strength; spatial proximity between nodes should be interpreted only as a layout aid. ICU networks (A) contain a peripheral Louvain community comprising colistin (COL), aztreonam (AZT), ceftazidime-avibactam (CZA), chloramphenicol (CMP), doxycycline (DOX), and tetracycline (TET), whereas in general wards (B) these agents are partitioned into separate peripheral communities (COL and CZA as singletons and AZT clustered with CMP and DOX). For interactive exploration, see Table S15, row 22 and row 21.

**Figure 10:**
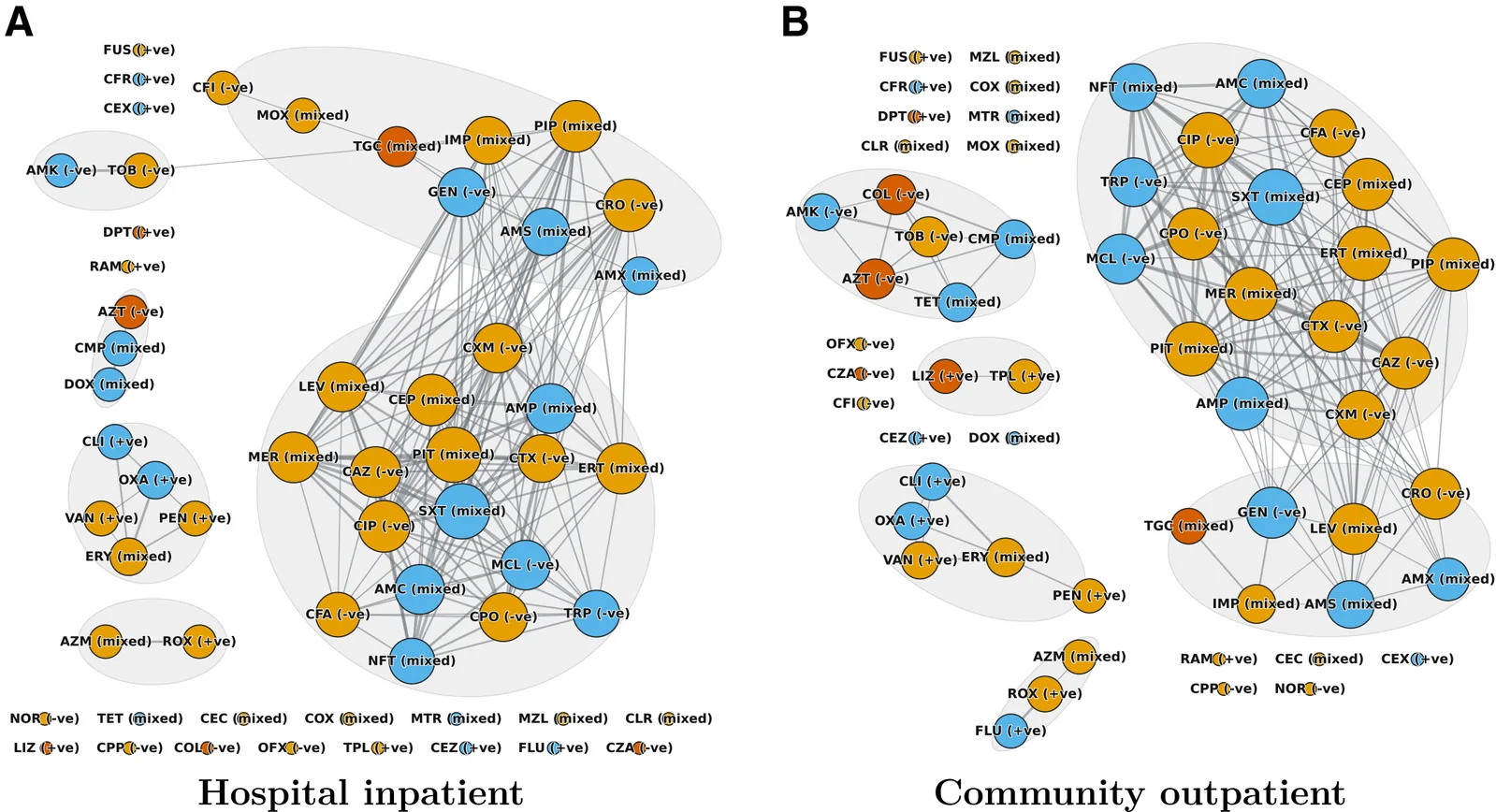
*Escherichia* urinary isolate co-testing networks stratified by care setting (hospital inpatient vs. community outpatient) Node color denotes WHO AWaRe class (blue, access; orange, watch; vermilion, reserve) and node size reflects testing prevalence. Edges represent Jaccard co-testing similarity (retained if *q* < 0.05), with thickness proportional to similarity strength; dashed envelopes denote Louvain communities. Layout note: node position in the force-directed layout reflects Louvain community assignment, not pairwise co-testing strength; spatial proximity between nodes should be interpreted only as a layout aid. Hospital inpatient networks (A) show an aminoglycoside community (amikacin [AMK]/tobramycin [TOB]) weakly bridged to the central testing core, which includes broad-spectrum beta-lactams (e.g., carbapenems, cephalosporins, and penicillins) and fluoroquinolones. Community outpatient networks (B) place aminoglycosides (AMK/TOB) within a peripheral community that also contains colistin (COL), aztreonam (AZT), chloramphenicol (CMP), and tetracycline (TET), separated from the central hub. For interactive exploration, see Table S15, row 20 and row 19.

#### Stage 2: Statistical validation of co-testing edges

Stage 2 assesses the statistical robustness of the best-performing configurations identified in stage 1. For the Jaccard-based networks defined by *τ*∗ = 0.30 and *γ*∗ = 1.0, FDR-corrected edge retention ranged from 89.2% (*Proteus*, blood culture) to 100.0% (*Escherichia*, urine) across the twelve organism-specimen combinations, with a median of 98.4%. Urine networks showed uniformly high retention (near or at 100%), whereas blood culture networks were slightly more variable (approximately 89%–100%).

Applying the same validation to the corresponding stage 1 optima of cosine, Dice, and *ϕ* yielded median retention of 98.7%, 98.8%, and 98.4%, respectively, with *ϕ* networks retaining 100% of testable edges. However, *ϕ* networks were much sparser (median 42 testable edges, range 27–70) than their Jaccard counterparts (median 123, range 55–266), with cosine and Dice in between (medians 226 and 183, ranges 179–518 and 135–406). Thus, stage 2 shows that the structurally preferred Jaccard configurations also preserve a large number of statistically supported co-testing relationships, providing a favorable balance between network richness and error-controlled inference. Together, these two stages demonstrate that Jaccard similarity with *τ*∗ = 0.30, *γ*∗ = 1.0 is both structurally optimal and statistically robust for constructing co-testing networks. Detailed per-cohort summaries, including ICS components, optimal parameter choices, and edge-retention statistics, are provided in Figure 11.

**Figure 11:**
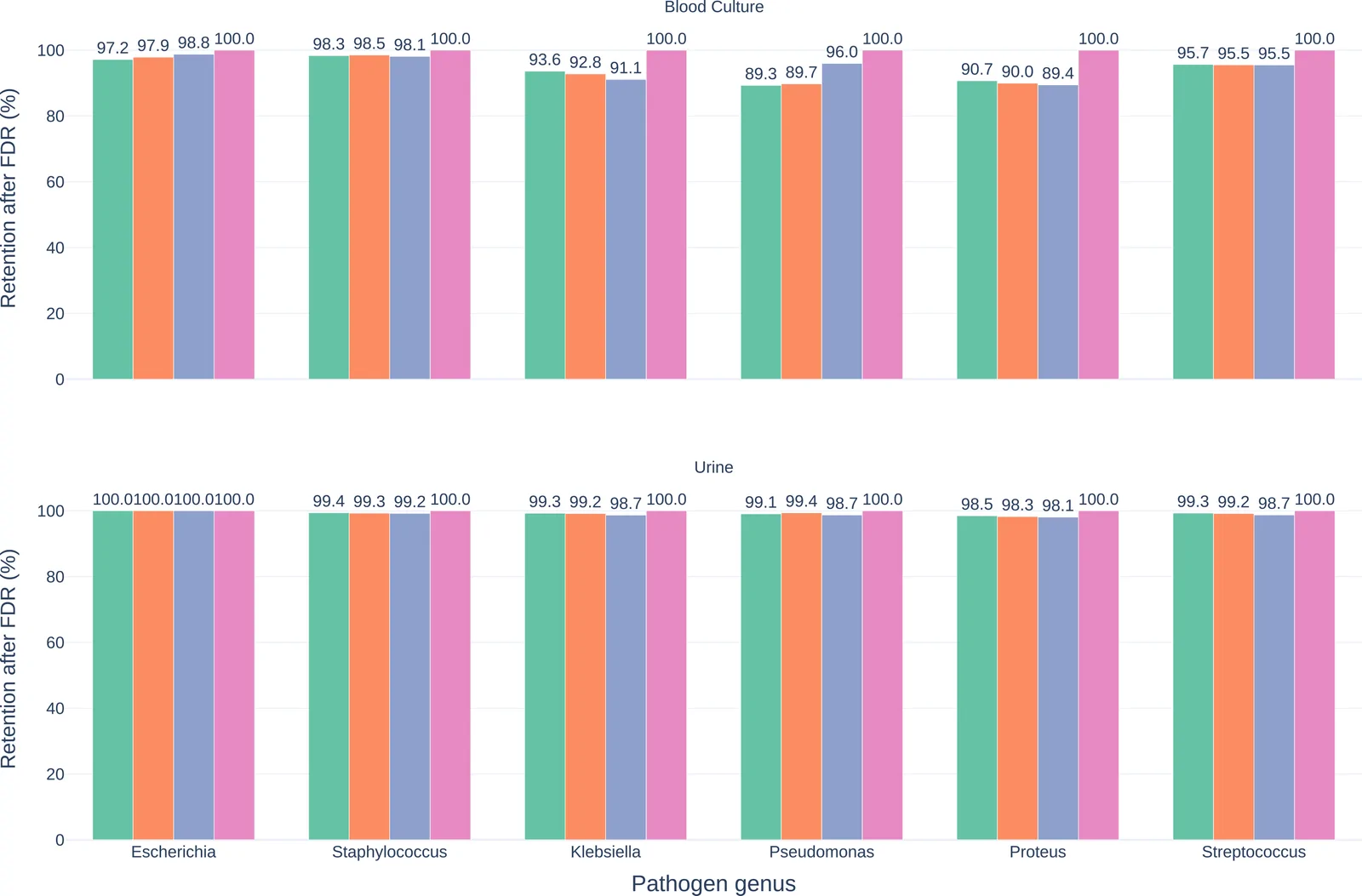
FDR-based retention of co-testing edges across pathogen cohorts and specimen types For each pathogen cohort (*Escherichia*, *Staphylococcus aureus*, *Klebsiella*, *Pseudomonas aeruginosa*, *Proteus*, and priority *Streptococcus*) and specimen type (blood culture and urine), the figure reports the proportion of Jaccard-derived edges (similarity ≥ *τ*∗ = 0.30) that remained statistically significant after Fisher’s exact test with Benjamini-Hochberg correction (FDR *α* = 0.05). Retention is calculated among testable edges only (i.e., pairs with sufficient joint observations to permit contingency testing). Across all cohort-specimen combinations, retention was high, demonstrating that the structural co-testing relationships identified by the similarity network are strongly supported by independent statistical evidence. Color note: colors represent distinct pathogen-specimen dataset combinations. They do not correspond to the WHO AWaRe classification scheme used in Figures 6, 7, 8, 9, and 10.

### Use case 1: Context-specific diagnostic strategies

To assess how clinical context reshapes antibiotic co-testing, we compared *Escherichia* networks across specimen types (urine vs. blood), ward acuity (ICU vs. general ward), and care settings (hospital vs. community). We define core communities as the standard, frequently co-tested antibiotics and “intermediate” communities as the “bridge” agents that link standard treatments to specialized peripheral/backup drugs. In this context, the term *agent* refers to an individual antibiotic drug that functions as a distinct node within the network. The variations in network composition and agent positioning across these contexts are summarized in Table 2, which highlights how different settings integrate core and intermediate drugs.

**Table 2:** Structural comparison of *Escherichia* antibiotic co-testing networks across specimen types and clinical setting**s**

Specimen	Setting	Core	Intermed.	Key agent behavior
Urine	ICU	13	10–11	colistin in cohesive periphery.
	ward	14	9	aminoglycosides bridged to core.
	outpatient	15	6–7	aminoglycosides isolated/disconnected.
Blood	ICU	16	11	colistin integrated into bridge.
	ward	15–17	5–6	nitrofurantoin relegated to periphery.
	outpatient	16	11	nitrofurantoin moved to bridge.
Universal	all	15–20	–	Gram-positive agents always peripheral.

This summary details core and intermediate community sizes and the relative positioning of specialized agents within each context.

The core group for urinary infections remains remarkably stable across all settings, anchored by essentials such as *nitrofurantoin*, *meropenem*, and *trimethoprim*. The primary distinction between settings lies in how backup drugs are organized. In the ICU, the network is highly integrated, keeping reserve agents like *colistin* and *aztreonam* in cohesive, ready-to-use groups. Conversely, in hospital wards and community settings, this structure loosens; backup drugs, and occasionally, *aminoglycosides*, become isolated and disconnected from the primary treatment bridge.

Blood culture networks displayed context dependence in their organization (Figures 7 and 8). These networks utilize a larger core of 18–20 broad-spectrum agents, including *β*-lactams and carbapenems. Unlike urine, the intermediate bridge in blood networks is highly flexible. In the ICU and community settings, this bridge expands significantly to incorporate backup drugs and even urinary-specific agents. In general hospital wards, however, this transition group shrinks to only five agents, leaving reserve drugs segregated on the periphery.

In all contexts, drugs intended for treating Gram-positive infections, such as *vancomycin* and *clindamycin*, remain on the extreme periphery. These combinations have limited or no valid clinical interpretability for the analyzed *Escherichia* cohort, which was overwhelmingly composed of *Escherichia coli*. Furthermore, the use of *tigecycline* increases directly with ward acuity, while nitrofurantoin, usually reserved for urine, frequently appears in blood culture networks, particularly in ICUs and community settings.

The organization of antibiotic co-testing networks across all contexts reflects a balance between standard treatments and reserve agents, as reflected in urine and blood culture networks. The distinction between settings lies in the intermediate communities: high-acuity settings, such as ICUs, maintain integrated networks, whereas community settings tend to isolate specialized drugs. The shape of these networks is influenced by clinical routine and institutional protocols as much as by biological necessity.

### Use case 2: Temporal evolution of diagnostic practice (2019–2023)

Across years, *E. coli* urine co-testing networks retained a large, densely connected core community with limited variation in overall structure (Figure 6, top row). In contrast, blood culture networks exhibited greater year-to-year variability, with more pronounced modular organization and shifts in peripheral communities (Figure 6, bottom row).

## Discussion

This study introduces the concept of the “SA”: the empirically observed networks of antibiotic co-testing decisions that underpin routine AMR surveillance. Using German national-scale AMR surveillance (ARS) data from 2019 to 2023, we reproduce and visualize the structural patterns of antimicrobial testing behavior through network analysis and community detection. To the best of our knowledge, this is one of the first studies to use a benchmarked, statistically filtered network framework to characterize diagnostic co-testing patterns on the scale of a national AMR surveillance system. It is also one of the first studies to operationalize the observable testing architecture underlying MNAR surveillance bias as a contextually stratified network property. As expected, the co-testing patterns appear to be context-sensitive rather than incidental. The central conceptual implication is that AMR surveillance data reflect both the epidemiology of microbial resistance and the underlying architecture of testing decisions, determining which antibiotics are tested and in which combinations and clinical contexts. Consequently, observed resistance patterns are influenced by an upstream decision-making process that is usually only implicit in conventional surveillance summaries. This work reveals testing patterns that were previously hidden and determines which resistance outcomes are observable in surveillance data.

A key methodological contribution of this work is its systematic evaluation of similarity measures for reconstructing co-testing structures from binary test incidence data. While using the *ϕ* coefficient produced the highest intrinsic coherence scores, it resulted in relatively small community sizes, making broader testing patterns more difficult to identify in the data. This behavior reflects the fact that, of all the similarity measures considered, the coefficient of *ϕ* is the only one sensitive enough to detect joint absences of test results. This makes the coefficient more selective about what it considers similar than the other measures. While this is advantageous for detecting highly specific conditional testing dependencies, it makes the coefficient less suitable for recovering integrated, panel-based testing practices. By contrast, Jaccard similarity produced a more parsimonious and operationally recognizable community structure of routine testing behavior, normalizing shared co-tests against overall testing exposure. In our analyses, this resulted in compact, reproducible communities that mostly aligned with established antibiotic classes. This methodological choice is supported by the successful recovery of context-invariant blood culture co-testing patterns in all contexts. Together, these findings suggest that Jaccard similarity is a sensible default method for constructing SAs when the objective is to characterize dominant co-testing patterns rather than rare or conditional combinations of tests.

The SA framework uses theoretical methods for network abstraction and community detection to summarize patterns of co-testing behavior. Its interpretation must therefore be considered in light of the modeling choices employed in these methods. Notably, the Louvain algorithm produces a non-overlapping partition of the antibiotics, assigning each one to a single community. This necessarily oversimplifies reality, as individual antibiotics may be part of multiple relevant co-testing communities. For example, different commercial vendors may offer different AST panels for their automated systems. However, this was a deliberate modeling choice, as our work aimed to identify the dominant empirical organization of testing practice as observed on a large scale rather than list all possible or hypothetical configurations. Variation in testing patterns in different contexts demonstrates that the framework can capture both invariant and context-dependent co-testing behavior: for example, there is temporal stability in blood culture testing patterns, but systematic reconfiguration of urine sample testing patterns across contexts. However, the fact that the Louvain algorithm generates a partition in which each antibiotic belongs to only one community may under-represent the operational bridge role of certain broad-spectrum agents. Agents such as carbapenems (meropenem, imipenem, and ertapenem), piperacillin-tazobactam, and ceftriaxone often appear in broad empirical-testing contexts and AST panel structures that span multiple suspected infection syndromes. This can cause them to connect otherwise distinct antimicrobial communities. However, the Louvain partition assigns each agent to a single community, suppressing this cross-community connectivity. Consequently, the framework may systematically underrepresent the secondary roles that core agents play in linking otherwise distinct testing communities. Alternative approaches, such as overlapping community detection (e.g., the Clique Percolation Method), mixed-membership stochastic block models, or hypergraph representations, could model multi-panel participation more explicitly, and are an important area for future research. However, their additional computational burden currently limits their scalability for national surveillance datasets of the size analyzed here. Nevertheless, they should be prioritized as methodological extensions.

Network visualizations use a force-directed layout to represent Louvain communities, where node position indicates community membership and edge thickness indicates Jaccard similarity (co-testing strength). Hospital inpatient networks exhibit dense central hubs with thick edges (high co-testing), whereas community networks exhibit sparse structures with isolated agent compartments (reduced co-testing). When working with the visualizations to explore co-testing patterns, it is important to bear in mind that spatial proximity represents community assignment rather than pairwise co-testing strength, and that visual prominence may de-emphasize isolated agents such as aminoglycosides, which may be clinically significant. Quantitative metrics (e.g., co-testing frequency, Jaccard similarity, and community modularity) can help confirm the relevance of the visual interpretation.

The SA framework distinguishes between co-testing patterns that are stable across different contexts and those that adapt to the context. The observed co-testing patterns for *Escherichia* serve as an example of this distinction. Urine networks maintained consistent topological organization across the different contexts considered, with observed variations reflecting quantitative differences in agent composition rather than fundamental restructuring. This stability is reflected in consistent node membership, inter-community connectivity, and community segregation, and is explained by both biological and institutional factors operating in concert. From a biological standpoint, urinary tract infections typically involve high bacterial loads, supporting reliable quantitative cultures and, consequently, more standardized, context-independent AST panel assignment. The clinical syndrome is often apparent before culture results are available, further narrowing panel selection. By contrast, blood cultures involve lower bacterial loads, enrichment-dependent recovery, longer incubation times and greater syndrome heterogeneity. Blood culture bacteremia arises from a clinically heterogeneous spectrum ranging from community-acquired sepsis to ICU-associated polymicrobial infection. This spectrum leads to broader, more clinician-directed panel ordering, which varies substantially by care setting and patient acuity. Institutional automation defaults and cascade reporting overlay these biological realities. While the two factors are inseparable in observational surveillance data, neither alone is the sole explanation. The networks produced by the SA framework generally produce sensible patterns. For example, they separate agents active against Gram-negative and Gram-positive pathogens into different communities. For outpatient *Escherichia* urine samples, the two largest communities contained the most typical first- and second-line treatments recommended by clinical guidelines for uncomplicated urinary tract infections.

One of the main insights from our results is that co-testing networks reveal unexpected patterns on a population scale. For instance, nitrofurantoin appeared within blood culture networks despite being primarily indicated for urinary tract infections, suggesting that testing may be driven by standardized panel composition rather than clinical indication. Similarly, vancomycin, clindamycin, and erythromycin appeared as co-testing nodes despite having limited or no valid clinical interpretability for the analyzed *Escherichia* cohort, which in the final aggregated network dataset consisted of records labeled *Escherichia coli*. Such patterns may reflect automated panel configuration, local reporting defaults, coding/mapping artifacts, or insufficient pathogen-antibiotic validation at the laboratory information management system level. For current surveillance reporting practice, however, this represents only a theoretical concern, as surveillance data on antibiotic resistance are typically reported only for relevant pathogen-antibiotic combinations selected manually. It could nonetheless be helpful to apply pathogen-antibiotic logical validation matrices before performing analyses on large raw surveillance datasets, to support this manual selection step. These matrices could flag, suppress, or require expert review of inherently implausible combinations of pathogens and antibiotics. Routinely generated SAs could provide an empirical monitoring layer for identifying recurrent anomalies and informing updates to these validation rules.

SAs transform diagnostic testing architecture from an invisible background variable into an explicit and observable component of surveillance. This has immediate practical applications. Laboratories could construct networks from routine data to document their testing behavior, categorized by pathogen and specimen type. Year-on-year comparisons of network structure can identify shifts in testing patterns and enable changes to be visualized. Testing frequency varies substantially across clinical contexts, yet remains invisible in conventional resistance reports, potentially obscuring a critical source of bias. The SA is not a report on the prevalence of resistance and does not adjust resistance rates. Rather, it is a co-testing map of the diagnostic observation process, identifying which antibiotic result fields are jointly observed or selectively observed within pathogen-specimen contexts. By mapping its co-testing architecture, a surveillance system or laboratory can identify which antibiotic-pathogen combinations are selectively tested and which clinical settings produce stable versus variable testing panels. The present paper is limited to the empirical characterization of the testing structure.

The mathematical construction of the SA is detailed in the STAR Methods and Methods S2, including the binary test-incidence representation, pairwise co-testing contingency tables, Jaccard-weighted network edges (Equation S2), and aggregation rules. The Methods S2 also includes an illustrative example (Equations S3 and S4) showing how the Jaccard co-testing structure could inform a future observation-process model. Conceptually, the Jaccard matrix summarizes how strongly antibiotic result fields are jointly observed, relative to isolates where at least one of the two was tested; unlike the *ϕ* coefficient, it does not credit joint absence of testing as similarity. The focus of the present study is the co-testing architecture, rather than estimation of adjusted resistance prevalence or implementation of a resistance-outcome model.

Together, these findings establish the SA as a novel framework for stewardship and surveillance interpretability. By making the testing architecture measurable and comparable across settings and time, the framework establishes a vital connection between laboratory practice and resistance surveillance.

### Limitations of the study

This study identifies the static structure of testing panels as reflected in co-testing networks, revealing important patterns in how antibiotics are grouped across clinical contexts. However, several key limitations constrain the scope of inference. Firstly, the framework identifies the co-occurrence of test results but cannot infer the directionality or sequencing of testing decisions. It cannot distinguish between simultaneous testing and conditional or reflex testing strategies, such as escalating to second-line antibiotics following resistance to first-line agents. Secondly, while the present analyses focus on *Escherichia* within the German national surveillance system, the specific co-testing patterns reported here may not generalize to other pathogens with different intrinsic susceptibility profiles (e.g., *Staphylococcus aureus*) or to international healthcare systems with different testing conventions and regulatory standards. The framework itself is pathogen- and system-agnostic; its application to further organisms and settings is a direct extension of this work. Thirdly, interactive visualization tools are required to enable medical professionals and surveillance system experts to explore patterns across multiple stratifications and develop a deeper understanding of co-testing structures within their own data. Fourthly, isolates were included without patient-level de-duplication, as the analytical goal was to capture aggregate laboratory testing behavior across all contributing institutions rather than patient-level epidemiology. However, chronically infected patients and long-term ICU admissions undergoing repeated susceptibility testing with identical institutional panels may contribute multiple isolates to the same analytical context. This could inflate Jaccard edge weights for specific antibiotic pairs. This effect is of greatest concern in the peripheral communities of blood culture networks, where absolute co-testing frequencies are lower and a modest number of duplicate records could exert disproportionate influence on edge weight estimates. Future analyses with access to patient-level identifiers should therefore assess the robustness of peripheral network structure and community membership under patient-level de-duplication.

The SA framework offers a complementary perspective on AMR surveillance data. It helps to visualize and understand co-testing patterns, which influence the interpretation of observed resistance patterns in surveillance systems. The observable resistance proportions will depend on diagnostic decisions, which are in part made visible by the SA. Across six WHO BPPL-aligned operational cohorts, Jaccard-based community detection consistently revealed structured co-testing communities that are context-sensitive: urine co-testing networks demonstrated stable, reproducible topological organization across clinical contexts, whereas blood culture networks exhibited greater year-to-year variability and context-dependent modular structure. Resistance surveillance cannot be interpreted independently of the diagnostic sampling frames that generate it. The proposed framework makes this explicit and provides an empirical foundation for interpreting AMR surveillance data when testing is selective or potentially MNAR.

## Resource availability

### Lead contact

Requests for further information and resources should be directed to and will be fulfilled by the lead contact, Ebenezer Awotoro (awotoroe@rki.de).

### Materials availability

This study did not generate new materials.

### Data and code availability

• Aggregated co-testing contingency tables generated in this study are publicly available at https://github.com/Ebenco36/shadow-antibiogram (dataset folder) as of the date of publication. A formally archived version with a persistent DOI has been deposited at Mendeley Data under the (https://doi.org/10.17632/8ffg3jb2vt). The original isolate-level data originate from the German Antibiotic Resistance Surveillance system (ARS); due to the terms of participation, these data cannot be shared publicly without approval from the contributing laboratories and are available from the ARS team at https://amr.rki.de/. All other data reported in this paper will be shared by the lead contact upon request.
• All original code has been deposited at GitHub under https://github.com/Ebenco36/shadow-antibiogram and is publicly available as of the date of publication.
• Any additional information required to reanalyze the data reported in this paper is available from the lead contact upon request.

## Acknowledgments

We would like to thank all the laboratories participating in the ARS surveillance system for contributing data to this analysis from 2019 to 2023.

## Author contributions

Conceptualization, E.A. and G.H.; methodology, E.A. and G.H.; software, E.A.; validation, A.A. and G.H.; investigation, E.A., N.W., and G.H.; resources, N.W. and G.H.; data curation, E.A.; writing – original draft, E.A.; writing – review and editing, A.A., C.A., N.W., and G.H.; visualization, E.A. and A.A.; supervision, G.H.; project administration, G.H.; funding acquisition, G.H. Funding: this research received no external funding.

## Declaration of interests

The authors declare no competing interests.

## STAR★Methods

### Key resources table

**Table:** 

REAGENT or RESOURCE	SOURCE	IDENTIFIER
**Deposited data**		

Aggregated co-testing contingency tables	This paper	https://github.com/Ebenco36/shadow-antibiogram/tree/main/datasets
Aggregated co-testing contingency tables (archived, persistent DOI)	This paper	Mendeley Data. https://doi.org/10.17632/8ffg3jb2vt
Original isolate-level ARS surveillance data (restricted, not publicly available)	Robert Koch-Institut^34^	https://amr.rki.de/
Supplemental sensitivity-analysis outputs for metric and parameter selection	This paper	https://github.com/Ebenco36/shadow-antibiogram/tree/main/sensitivity_result

**Software and algorithms**		

Python, version 3.12.10	Python Software Foundation^35^	https://www.python.org/
pandas, version 2.3.0	McKinney^36^	https://pandas.pydata.org/
NumPy, version 1.26.4	Harris.et al.^37^	https://numpy.org/
PyArrow, version 21.0.0	Apache Software Foundation^38^	https://arrow.apache.org/docs/python/
NetworkX, version 3.5	Hagberg.et al.^39^	https://networkx.org/
python-louvain, version 0.16	Blondel.et al.^40^	https://github.com/taynaud/python-louvain; https://doi.org/10.1088/1742-5468/2008/10/P10008
SciPy, version 1.13.1	Virtanen.et al.^41^	https://scipy.org/
statsmodels, version 0.14.5	Seabold, S., Perktold, J.^42^	https://www.statsmodels.org/
scikit-learn, version 1.5.2	Pedregosa.et al.^43^	https://scikit-learn.org
Matplotlib, version 3.10.5	Hunter^44^	https://matplotlib.org/
Plotly, version 6.0.0	Plotly Technologies Inc^45^	https://plotly.com/python
Custom Shadow Antibiogram analysis pipeline	This paper	https://github.com/Ebenco36/shadow-antibiogram

**Other**		

German Antimicrobial Resistance Surveillance system (ARS)	Robert Koch-Institut^34^	https://amr.rki.de/
WHO AWaRe antibiotic classification, 2023 edition	World Health Organization^8^	https://www.who.int/publications/i/item/WHO-MHP-HPS-EML-2023.04
WHO Bacterial Priority Pathogens List, 2024	World Health Organization^9^	https://www.who.int/publications/i/item/9789240093461; https://iris.who.int/server/api/core/bitstreams/1a41ef7e-dd24-4ce6-a9a6-1573562e7f37/content
DrugBank database	DrugBank^46^	https://go.drugbank.com/
EUCAST clinical breakpoints	European Committee on Antimicrobial Susceptibility Testing^47^	https://www.eucast.org/

### Experimental model and study participant details

This study does not involve experimental models, cell lines, or animal subjects. All analyses were performed on de-identified routine clinical surveillance records; further details are provided in method details (Ethics and Data source).

### Method details

#### Ethics

The study analyzed anonymized routine surveillance data from the Robert Koch Institute (RKI). Analysis of de-identified surveillance data is exempt under German surveillance law (§13 IfSG) and Article 89 GDPR.^48^^,^^49^

#### Data source

We analyzed antimicrobial susceptibility testing (AST) data from the national Antibiotic Resistance Surveillance (ARS) system in Germany, which is coordinated by the Robert Koch Institute (RKI). Established in 2008, ARS is one of Europe’s largest surveillance networks for AMR, incorporating data from hospital and outpatient laboratories in all 16 German federal states.^50^^,^^51^ By 2020, over 550 laboratories had contributed data at the level of individual isolates, from a variety of care settings and regional contexts.^51^ Laboratories submit routine diagnostic results electronically with selected metainformation, including information on the specimen and isolated pathogens, selected patient demographics and organization context of specimen collection (e.g., hospital/medical practice, ward type of patient if in hospital, geographical location). AST results are reported as categorical interpretations (susceptible, intermediate or resistant) according to clinical breakpoints defined by the laboratory standard (e.g., EUCAST).^47^ The study period runs from 1 January 2019 to 31 December 2023 and comprises 13 million clinical isolates.

#### Data preprocessing and quality control

We implemented a multi-stage preprocessing pipeline to standardize the data and ensure reproducibility. All analyses were conducted on both the complete isolate-level dataset and the aggregated dataset to verify that the aggregation preserved the findings. For public release and replication, we provide aggregated contingency tables of co-testing patterns within predefined strata, ensuring reproducibility of the presented co-testing networks (see Section S5).

#### Data standardization and harmonization

To ensure analytical consistency and interpretability, age was standardized into seven analytical strata (<15, 15–29, 30–44, 45–59, 60–74, 75–89, ≥90 years) to balance statistical resolution with clinically meaningful age distributions (Table S9). Other demographic, clinical, and laboratory variables were similarly standardized for consistency across analyses.

Sex was coded as male, female, other/unknown. Specimen types were standardized into clinically relevant categories: urine; wound; swab; blood culture; respiratory; and others. The care setting was standardized as inpatient, outpatient, or other. Ward/unit type was coded to distinguish intensive care units (ICUs) from general wards (non-ICU) settings. Pathogen names were validated against current taxonomic nomenclature, including reconciliation of alternative and German-language designations. Antimicrobial agent names were reconciled to a standardized reference classification documenting drug class and WHO AWaRe category.

#### Recording and antibiotic filtering strategy

In order to focus on the most relevant antibiotics, we only considered substances that had been tested on at least 1% of the isolates. This approach retained 58 antimicrobial agents for analysis. We further restricted inclusion to agents listed in the WHO AWaRe (Access, Watch, Reserve) classification (2023 edition)^8^ and considered only isolates belonging to WHO BPPL-listed or BPPL-aligned operational taxonomic cohorts.^9^ Pathogen cohorts were defined as WHO BPPL-aligned operational surveillance cohorts. Because the present study characterizes testing architecture rather than resistance outcomes, BPPL categories were used to define taxonomic surveillance cohorts, not resistance-phenotype-restricted cohorts. The WHO BPPL 2024 lists resistant Enterobacterales as a critical-priority group; this broad category was operationalized in ARS by selecting consistently reported Enterobacterales genera, including *Escherichia*, *Klebsiella*, *Proteus*, *Enterobacter*, *Citrobacter*, *Serratia*, *Morganella*, and *Providencia*. Species-level cohorts were retained where both the BPPL target and ARS reporting consistently supported species-level identification (*Staphylococcus aureus*, *Pseudomonas aeruginosa*). For streptococci, the BPPL medium-priority entries include Group A Streptococci, *Streptococcus pneumoniae*, and Group B Streptococci, which were analyzed as a priority *Streptococcus* cohort. This broad set of BPPL-aligned taxa formed the study dataset; for focused network construction, six high-volume operational cohorts were selected: *Escherichia* spp., *Klebsiella* spp., *Proteus* spp., *Staphylococcus aureus*, *Pseudomonas aeruginosa*, and the priority *Streptococcus* cohort. Included isolates needed to be tested for at least one antibiotic. Isolates with missing data on material type and care setting were omitted from the study dataset. Tables S5–S7 document testing frequencies and filtering outcomes at each step. Figure 2 illustrates the complete filtering strategy from raw records to the final analytical dataset.

#### Test-incidence encoding and domain annotation

A binary test-incidence matrix *X* represents antimicrobial testing patterns, where an entry *X*_*ji*_ indicates whether susceptibility testing was performed for antibiotic *i* on isolate *j*. Specifically:

(Equation 1) $$X_{ji}=\left\{\begin{array}{cc} 1, & \text{if}\;\text{antibiotic}\;i\;\text{was}\;\text{tested}\;\text{for}\;\text{isolate}\;j,\\ 0, & \text{if}\;\text{antibiotic}\;i\;\text{was}\;\text{not}\;\text{tested.} \end{array}\right)$$

Each isolate is represented by a binary vector *X*_*j*⋅_ of length *A*, where *A* denotes the total number of antibiotics retained after filtering across the entire dataset. These patterns reflect the behavior of laboratory testing and diagnostic decisions in each isolate-specific context.

To facilitate clinical interpretation, each antibiotic was annotated as follows: (i) the WHO AWaRe category (Access, Watch or Reserve) (WHO, 2023); (ii) the pharmacological class (penicillin, cephalosporin, carbapenem, fluoroquinolone, aminoglycoside, macrolide, tetracycline, glycopeptide, oxazolidinone, polymyxin, nitrofuran or folate-pathway inhibitor); and (iii) the target spectrum of activity (Gram-positive, Gram-negative or mixed/broad), extracted from the DrugBank database.^46^ Pathogens were annotated based on Gram stain (positive, negative or other), taxonomic identification (genus and species, if available) and their priority level on the WHO Bacterial Priority Pathogens List (critical, high, medium or unlisted).^9^ Table 2 provides an example of isolate records with patient attributes (age group, sex), diagnostic setting (specimen type, care setting, ward type), and temporal information (calendar year).

#### Analytical framework and context stratification

Building on the binary test-incidence representation, which captures decisions about presence or absence of a test results in the data (diagnostic inclusion), we analyzed co-testing behavior in different clinical contexts. Each context g∈S was specified as a unique combination of pathogen (genus or species), specimen type, care setting, and ward type. Analyses were primarily conducted at the context-year level (*t* ∈ {2019, *…*, 2023}), with the option of aggregation or comparison across broader temporal or contextual resolutions.

For each analytical unit, we constructed a binary test-incidence matrix *X* ∈ {0,1}^*N*×*A*^ covering all considered antibiotics (*A* = 58), where *N* denotes the number of isolates within the subset (e.g., a given context-year), and *X*_*ji*_ = 1 indicates that antibiotic *i* was tested for isolate *j*. This matrix captures the diagnostic panel composition applied to each isolate and provides a standardized basis for quantifying co-testing behavior and inferring diagnostic network structure. The complete analytical workflow, integrating cohort preparation, similarity metric optimization and community detection, is detailed in Figure S1.

#### Network construction and the Shadow Antibiogram framework

Building on the previously described statistical filtering, we modeled each diagnostic context as an undirected, weighted network representing observed antibiotic co-testing behavior. For each context or context-year combination, we constructed networks where nodes represent antibiotics and edge weights reflect the strength of co-testing associations in routine practice (see supplemental information, Section S2). Each network provides an empirical snapshot of co-testing patterns within a given clinical context, aggregated across all contributing organizations rather than representing any single institutional protocol. These networks form an ensemble across pathogens, specimen types, care settings, and time periods, enabling systematic comparison of co-testing patterns.

#### Community detection via louvain clustering

The Louvain algorithm^40^ was used to identify communities within each network, grouping antibiotics that are more densely connected to each other than to the rest of the network through modularity optimization. This approach is computationally efficient for large, sparse, weighted networks, enabling scalable community detection on co-testing graphs with thousands of nodes and many edges, where more computationally intensive approaches would be impractical. The resolution parameter was fixed at *γ* = 1.0 (established as optimal in preliminary sensitivity analyses; Supplemental Table^33^; Section 3). For all subsequent analyses, 100 independent Louvain initializations were performed for each network to assess partition reproducibility. Within this framework, the Shadow Antibiogram is defined as the set of latent communities (clusters of antibiotics routinely co-tested) revealed by empirical practice rather than formal guidelines. For each context, we identify communities representing recurrent antibiotic combinations tested together for shared diagnostic or therapeutic purposes (see supplemental information, Section S2).

#### Intrinsic evaluation and selection of similarity metrics

We evaluated four binary association measures (Jaccard, Dice, Cosine, and *ϕ*) using a two-stage framework: Stage 1 identified the metric and parameters yielding the most stable and coherent networks; Stage 2 validated edges using formal statistical testing. This separation prevents p-value-driven bias in metric selection.

##### Stage 1: Intrinsic network-quality-driven metric selection

For each pathogen-specimen subset, pairwise similarity matrices were computed over a parameter grid: edge-weight threshold *τ* ∈ {0.10, 0.20, 0.30, 0.40} and Louvain resolution *γ* ∈ {0.50, 0.75, 1.00}, with 100 independent runs per combination. Metrics were evaluated on three criteria: (1) Community stability (mean Adjusted Rand Index across partitions)^52^ and internal coherence (Intrinsic Coherence Score, defined as the ratio of mean within-community to global mean similarity; ICS >1.0 indicates genuine co-testing signal); (2) Alignment with hierarchical antimicrobial classifications (fine classes, broad classes, WHO AWaRe) using Normalized Mutual Information^53^ with weights (0.40, 0.30, 0.30), and label-free community separation using silhouette scores^54^; (3) Joint maximization of stability, ICS, biological alignment, and silhouette cohesion across pathogen cohorts, yielding consensus parameters *τ*∗ = 0.30, *γ*∗ = 1.0. Detailed methods are in Section S3.

##### Stage 2: Post-hoc statistical validation of co-testing edges

For each antibiotic pair with similarity greater than or equal to *τ*∗, Fisher’s exact test was applied to a 2 × 2 contingency table:

$$\left[\begin{array}{cc} n_{11} & n_{10}\\ n_{01} & n_{00} \end{array}\right],\;p=Fisher\left(n_{11},n_{10},n_{01},n_{00}\right),$$

where *n*_11_ denotes joint testing, *n*_10_ and *n*_01_ unilateral testing, and *n*_00_ neither tested. *p*-values were adjusted using Benjamini-Hochberg (FDR, *α* = 0.05); edges retained if *q* ≤ *α*. This ensures final networks satisfy structural (stable, coherent, biologically aligned communities) and statistical (significant co-testing associations) validity.

#### Analyses and use cases

Two use cases guided our analysis: how co-testing patterns change over time and how they vary based on context.

##### Use case 1: Context-specific diagnostic strategies

To assess whether diagnostic behavior varies across clinical contexts, we constructed paired co-testing networks for predefined dichotomies spanning care setting, institutional complexity, and patient characteristics. We define a *cohort* as a group of isolates meeting specific criteria (pathogen genus, specimen type, year, care setting), and a *context* as a single dimension along which cohorts differ (e.g., ICU versus general ward, inpatient versus outpatient). Each paired comparison consisted of two non-overlapping cohorts matched on pathogen genus, specimen type, and antibiotic panel, differing only in a single contextual dimension (e.g., ICU versus general ward, inpatient versus outpatient care, tertiary versus primary/secondary facilities, or sex in urinary isolates). Each cohort required a minimum of 1,000 isolates to ensure robust network reconstruction. For all cohort comparisons, all isolates meeting the cohort definition were included without de-duplication by patient. This approach captures the full diagnostic testing behavior across each population without imposing patient-level restrictions, reflecting real-world laboratory and surveillance practice. Isolates were required to have at least one recorded susceptibility test among the retained antibiotics, while untested antibiotic fields were preserved as part of the test-incidence structure.

All networks used the globally optimized Jaccard configuration (*τ*∗ = 0.30, *γ*∗ = 1.0), with edges retained only if supported by Fisher’s exact test with Benjamini-Hochberg correction (*q* ≤ 0.05), and Louvain communities obtained under the same parameters. Differences between contexts were examined by comparing the organization of antibiotic communities, the presence or absence of key co-testing links, and the extent to which testing panels appeared broad, targeted, modular or diffuse. As modeling choices were held constant, structural differences could be interpreted as divergence in diagnostic strategy rather than methodological artifacts. This enabled context-aware interpretation of co-testing behavior across care settings, patient groups and levels of clinical acuity.

##### Use case 2: Temporal evolution of diagnostic practice (2019–2023) – Confirmation of network reproducibility

To confirm the reproducibility of co-testing patterns, we constructed antibiotic co-testing networks for *Escherichia* in urine and blood culture specimens based on aggregated yearly data from 2019 to 2023. Cohorts with fewer than 1,000 isolates were excluded to ensure sufficient support for pairwise co-testing frequencies, and antibiotics that were never tested within a given year were removed. Temporal evolution was assessed by comparing annual network structure, including shifts in connectivity, emergence or dissolution of diagnostic communities, and changes in community modularity across years.

### Quantification and statistical analysis

#### Co-testing quantification and pairwise association metrics

Within each analytical context or context-year pair, pairwise co-testing between antibiotics *i* and *k* was quantified as the number of isolates for which both were tested:

(Equation 2) $$c_{ik}=\underset{j\in J}{\sum }X_{ji}X_{jk},$$

where J denotes the set of isolates in the analytical subset and N=|J|; with the marginal testing frequencies as:

(Equation 3) $$n_{i}=\underset{j\in J}{\sum }X_{ji},\;n_{k}=\underset{j\in J}{\sum }X_{jk}.$$

These quantities form a 2 × 2 contingency table (Table S1). Four binary association metrics were computed to quantify co-testing strength: Jaccard index,^55^^,^^56^^,^^57^ Dice coefficient,^57^^,^^58^ Cosine similarity,^57^^,^^59^ and Phi coefficient (*ϕ*).^57^^,^^59^^,^^60^ Their comparative performance is evaluated in Section.

#### Statistical significance and multiple-testing control

To distinguish systematic co-testing relationships from random co-occurrence, we applied Fisher’s exact test to each antibiotic pair (*i*, *k*) within a given analytical/cohort data-specific subset. Resulting *p*-values were corrected for multiple comparisons using the Benjamini–Hochberg (BH) procedure,^61^ controlling the output of the BH procedure at *α* = 0.05. The retained set of pairs defines a sparse, symmetric, weighted adjacency matrix *W*, with entries *W*_*ik*_ = Jaccard_*ik*_ for retained pairs and *W*_*ik*_ = 0 otherwise.

## Supplemental information

Document S1. Figures S1–S3, Tables S1–S16, Methods S1–S5, Section S1–S5, and Equations S1–S6

Data S1. Bacterial Genera by treatment type

Data S2. AST volumes for major Gram-negative pathogens by antibiotic

Data S3. Antibiotic retained after coverage filtering

Data S4. Antibiotics excluded by coverage threshold

Data S5. Age and sex distribution by bacteria genus

Data S6. Bacterial genera by Gram-stain and specimen type

Data S7. Temporal cohort summary by specimen type and year
