# Supplementary material for: Network-based characterization of latent co-testing patterns in antimicrobial susceptibility testing
Source: iScience. 2026 Sep 22;29(10):117421. doi: 10.1016/j.isci.2026.117421 (PMC13632225; doi:10.1016/j.isci.2026.117421)
Supplement: Supplementary file 1 [file mmc1.pdf]

iScience, Volume *29*

Supplemental information

Network-based characterization of latent co-testing patterns in antimicrobial susceptibility testing

Ebenezer Awotoro, Aleksandar Anžel, Chisom Anyabolu, Niklas Willrich, and Georges Hattab

Network-based characterisation of latent co-testing patterns in antimicrobial susceptibility testing

Ebenezer Awotoro[1,2,*], Aleksandar Anžel[1], Chisom Anyabolu[1], Niklas Willrich[3], and Georges Hattab[1,2,**]

[1]Center for Artificial Intelligence in Public Health Research (ZKI-PH), Robert Koch Institute, 13353, Berlin, Germany
[2]Department of Mathematics and Computer Science, Freie Universität Berlin, 14195, Berlin, Germany
[3]Department of Infectious Disease Epidemiology, Robert Koch Institute, Berlin, Germany
[*]Lead contact: awotoroe@rki.de
[**]Correspondence: hattabg@rki.de

1 Contingency table structure for co-testing analysis

For antibiotics i and k:

Table S1: Contingency structure for co-testing between antibiotics i and k.

	Antibiotic k		
Antibiotic i	Tested	Not tested	Total
Tested	c_{ik}	$n_i - c_{ik}$	n_i
Not tested	$n_k - c_{ik}$	$N - n_i - n_k + c_{ik}$	$N - n_i$
Total	n_k	$N - n_k$	N

2 Network construction and the shadow antibiogram framework

For each stratum or stratum-year (g, t), we constructed

$$G_{g,t} = (V, E_{g,t}, W_{g,t}), \tag{S1}$$

where V is the retained set of antibiotics, $E_{g,t} = \{(i, k) : W_{ik} > 0\}$ denotes statistically significant co-testing pairs, and $W_{g,t}$ stores Jaccard weights for each edge.

The Jaccard co-testing weight for antibiotics i and k within stratum (g, t) is

$$J_{ik}^{(g,t)} = \frac{\sum_{j \in \mathcal{I}_{g,t}} X_{ji} X_{jk}}{\sum_{j \in \mathcal{I}_{g,t}} \mathbf{1}(X_{ji} + X_{jk} > 0)}, \tag{S2}$$

where $\mathcal{I}_{g,t}$ is the set of isolates in stratum (g, t), and $X_{ji} \in \{0, 1\}$ is the binary test-incidence indicator for antibiotic i on isolate j (defined in the Methods). The numerator equals c_{ik} and the denominator equals $n_i + n_k - c_{ik}$ from Table S1. For the retained networks used in the main analyses, $W_{ik} = J_{ik}^{(g,t)}$ only for pairs satisfying the Jaccard threshold ($J_{ik}^{(g,t)} \geq \tau^*$) and the corresponding Fisher's exact test with Benjamini–Hochberg correction ($q \leq 0.05$); otherwise, $W_{ik} = 0$.

2.1 Illustrative example: Jaccard information as an observation-process covariate

The present study does not estimate adjusted resistance prevalence, and the Jaccard co-testing matrix does not adjust resistance rates by itself. The equations below are included only as an illustrative future use case: Jaccard-derived co-testing information could be used as a covariate in a model for whether a resistance result is observed. Let R_{ji} denote the resistance status of isolate j to antibiotic i. In routine surveillance data, R_{ji} is observed only when $X_{ji} = 1$. Thus the observed resistance proportion,

$$P(R_{ji} = 1 \mid X_{ji} = 1, g, t),$$

need not equal the target resistance prevalence $P(R_{ji} = 1 \mid g, t)$ when testing is selective.

A future observation-process model could estimate

$$\pi_{ji} = P\left(X_{ji} = 1 \mid \mathbf{z}_j, \mathbf{J}_{i\cdot}^{(g,t)}\right), \tag{S3}$$

where $\mathbf{z}_j$ denotes isolate-level or context-level covariates, and $\mathbf{J}_{i\cdot}^{(g,t)}$ is the row of the Jaccard matrix summarising how antibiotic i is empirically coupled to other antibiotic test fields in stratum (g, t). Estimated testing probabilities could then be used in an inverse-probability-weighted estimator such as

$$\widehat{\rho}_i^{(g,t)} = \frac{\sum_{j \in \mathcal{I}_{g,t}} X_{ji} R_{ji} / \widehat{\pi}_{ji}}{\sum_{j \in \mathcal{I}_{g,t}} X_{ji} / \widehat{\pi}_{ji}}, \tag{S4}$$

where $\widehat{\rho}_i^{(g,t)}$ is a model-based estimate of resistance prevalence for antibiotic i in stratum (g, t). Valid use of such an estimator would require the usual assumptions for inverse-probability weighting, including a correctly specified observation model and positivity of the testing probability within the analysed stratum. This expression is provided only as an illustrative example of how co-testing structure could be used as input to future observation-process modelling: the analyses reported in the present paper stop at empirical characterisation of co-testing architecture and do not fit Equation S3 or Equation S4.

Together, these graphs form the ensemble

$$\mathcal{G} = \{G_{g,t} \mid g \in \mathcal{S},\ t \in \mathcal{T}\},$$

which enables a systematic comparison to be made across pathogens, specimen types, care settings, and time periods.

For each context (g, t), we identify a partition

$$C_{g,t} = \{C_1, C_2, \ldots, C_K\}, \tag{S5}$$

where each community C_k represents a recurrent panel of antibiotics that are tested together for shared diagnostic or therapeutic purposes.

3 Community detection via Louvain clustering

The Louvain algorithm was used to identify communities within each network $G_{g,t}$, which maximises network modularity, a principled measure of community structure.

The modularity objective is

$$Q(\gamma) = \frac{1}{2m} \sum_{i,k} \left(W_{ik} - \gamma \frac{s_i s_k}{2m} \right) \mathbf{1}_{\{c(i)=c(k)\}}, \tag{S6}$$

where $m = \frac{1}{2} \sum_{i,k} W_{ik}$ is total edge weight, $s_i = \sum_k W_{ik}$ is node strength, and $\mathbf{1}_{\{c(i)=c(k)\}}$ indicates community membership.

The resolution parameter was fixed at $\gamma = 1.0$. At this resolution, 100 independent Louvain initialisations were performed for each network to assess partition reproducibility.

3.1 Intrinsic evaluation and selection of similarity metrics

4 Stage 1: Intrinsic network-quality-driven metric selection

All similarity metrics were evaluated on their ability to produce stable, internally coherent and biologically aligned community structures without any statistical filtering based on the FDR-corrected statistical significances, so that metric selection reflects the intrinsic geometry of similarity patterns rather than artefacts of sample size.

For each pathogen-specimen subset, pairwise similarity matrices were computed over a grid of parameters $\tau \in \{0.10, 0.20, 0.30, 0.40\}$ (edge-weight threshold) and $\gamma \in \{0.50, 0.75, 1.00\}$ (Louvain resolution); for each (τ, γ) combination, Louvain was run $R = 100$ times with independent random seeds.

4.1 Clustering stability and Intrinsic Coherence Scores (ICS)

For each parameter pair (τ, γ) and similarity metric, Louvain produced partitions $\mathcal{P}_1, \ldots, \mathcal{P}_R$, and stability was quantified as the mean pairwise Adjusted Rand Index (ARI):

$$\mathrm{Stability} = \frac{2}{R(R-1)} \sum_{1 \le i < j \le R} \mathrm{ARI}(\mathcal{P}_i, \mathcal{P}_j),$$

where higher values indicate greater robustness of community structure.

Given a similarity matrix $S \in \mathbb{R}^{n \times n}$ over n antibiotics and a partition C, internal enrichment was measured by the Intrinsic Coherence Score,

$$\mathrm{ICS} = \frac{\mu_{\text{within}}}{\mu_{\text{global}}},$$

Table S2: Distribution of bacterial genera in surveillance dataset (2019–2023).

Bacterial Genus	Count	Percentage
Escherichia	4,728,867	36.4%
Staphylococcus	2,138,013	16.5%
Klebsiella	1,585,973	12.2%
Pseudomonas	974,462	7.5%
Proteus	928,415	7.1%
Streptococcus	658,597	5.1%
Enterobacter	492,088	3.8%
Enterococcus	483,146	3.7%
Citrobacter	348,531	2.7%
Serratia	205,655	1.6%
Morganella	167,003	1.3%
Haemophilus	133,286	1.0%
Acinetobacter	86,187	0.7%
Providencia	42,392	0.3%
Mycobacterium	8,380	0.1%

where μ_{global} is the mean similarity across all unordered pairs and μ_{within} the mean similarity among pairs assigned to the same community; values $\mathrm{ICS} > 1$ indicate within-community similarities exceed the global background.

4.2 External and label-free alignment

Each partition was compared against hierarchical antimicrobial classifications (fine classes, broad classes, and WHO AWaRe) using Normalised Mutual Information (NMI), and a composite score

$$0.40\,\mathrm{NMI}_{\text{fine}} + 0.30\,\mathrm{NMI}_{\text{broad}} + 0.30\,\mathrm{NMI}_{\text{WHO}},$$

where the weights (0.40, 0.30, 0.30) reflect the clinical primacy of fine-grained drug classification over broader categories.

In parallel, silhouette scores were computed from the dissimilarity matrix $D = 1 - S$ to quantify cluster separation independently of external labels.

4.3 Optimal (τ, γ) selection

For each metric and pathogen genus, an optimal parameter pair (τ^*, γ^*) was selected by jointly maximising clustering stability, ICS, biological alignment, and silhouette cohesion. Median aggregation across genera yielded the consensus configuration

$$\tau^* = 0.30, \quad \gamma^* = 1.0,$$

which was fixed for all downstream analyses, including temporal dynamics, context-specific divergence, and statistical validation.

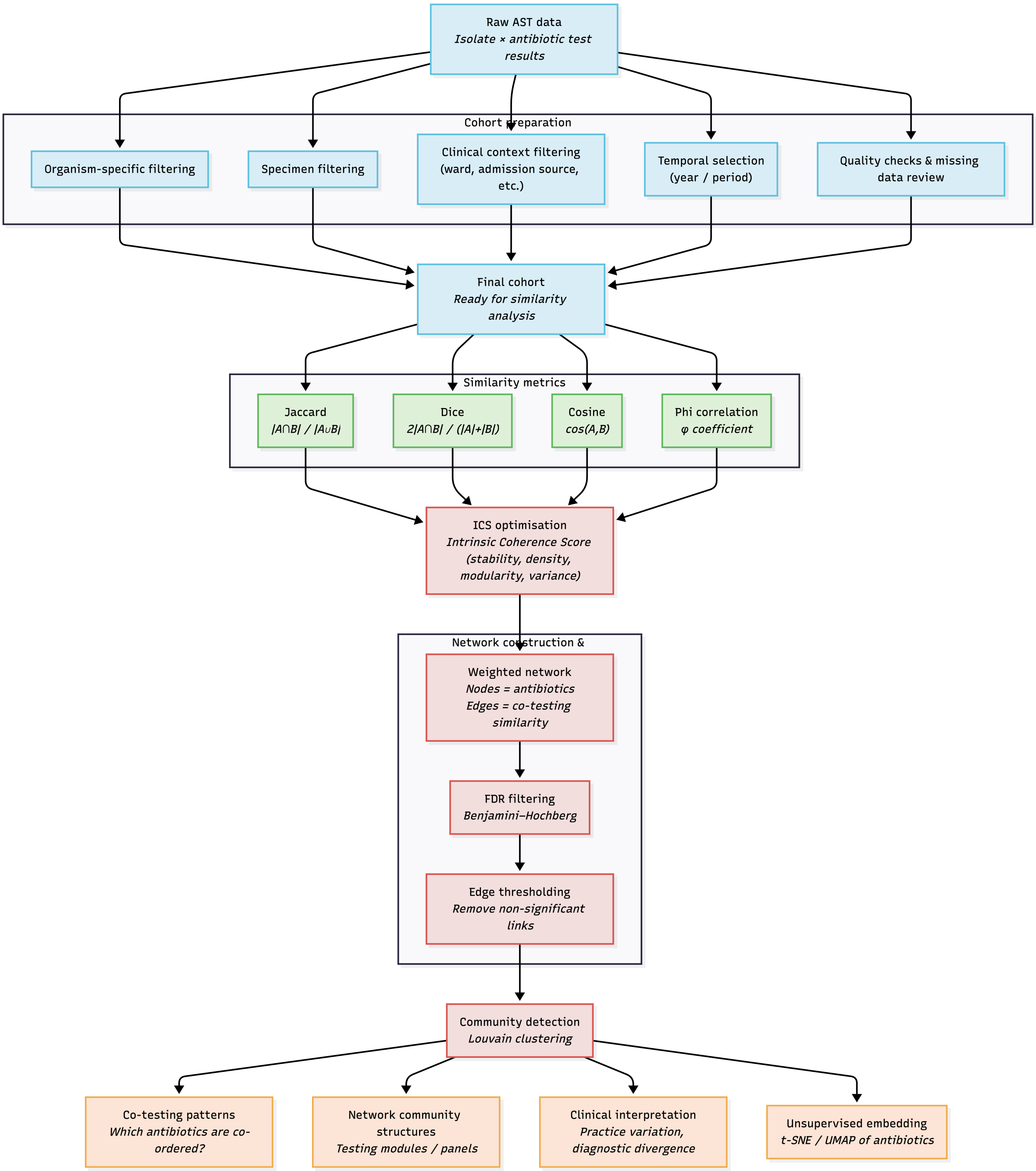

Figure S1: This workflow illustrates the end-to-end methodological framework, proceeding from raw AST data extraction to network inference. Top panel: Cohort preparation steps corresponding to the multi-stage filtering strategy (Section 2.3). Middle panel: The intrinsic evaluation framework used to select the optimal similarity metric via Intrinsic Coherence Score (ICS) optimization (Section 2.10). Bottom panel: Network construction, including False Discovery Rate (FDR) filtering (Section 2.7), edge thresholding, and Louvain community detection (Section 2.9). Key mathematical definitions for the evaluated similarity metrics (Jaccard, Dice, Cosine, Phi) are displayed in the green modules.

Table S3: Distribution of specimen material types (2019–2023).

Specimen Material	Count	Percentage
Urine	6,009,092	46.3%
Swab	2,148,484	16.5%
Wound	2,120,392	16.3%
Respiratory	766,231	5.9%
Blood Culture	626,129	4.8%
Others	494,644	3.8%
Urogenital Swab	476,889	3.7%
Punctate	310,768	2.4%
Stool Samples	37,942	0.3%
Exclusion	86	0.0%

Table S4: Patient age distribution of bacterial isolates (2019–2023).

Age Range	Count	Percentage
80–84 years	1,853,526	14.3%
75–79 years	1,481,215	11.4%
70–74 years	1,304,229	10.0%
85–89 years	1,223,636	9.4%
65–69 years	1,183,429	9.1%
60–64 years	1,008,582	7.8%
55–59 years	812,285	6.3%
50–54 years	555,570	4.3%
90–94 years	509,264	3.9%
45–49 years	359,050	2.8%
0 years	345,421	2.7%
30–34 years	343,253	2.6%
35–39 years	340,292	2.6%
40–44 years	322,551	2.5%
25–29 years	280,875	2.2%
20–24 years	242,117	1.9%
1–4 years	222,811	1.7%
15–19 years	184,714	1.4%
5–9 years	171,176	1.3%
≥95 years	127,988	1.0%
10–14 years	111,373	0.9%

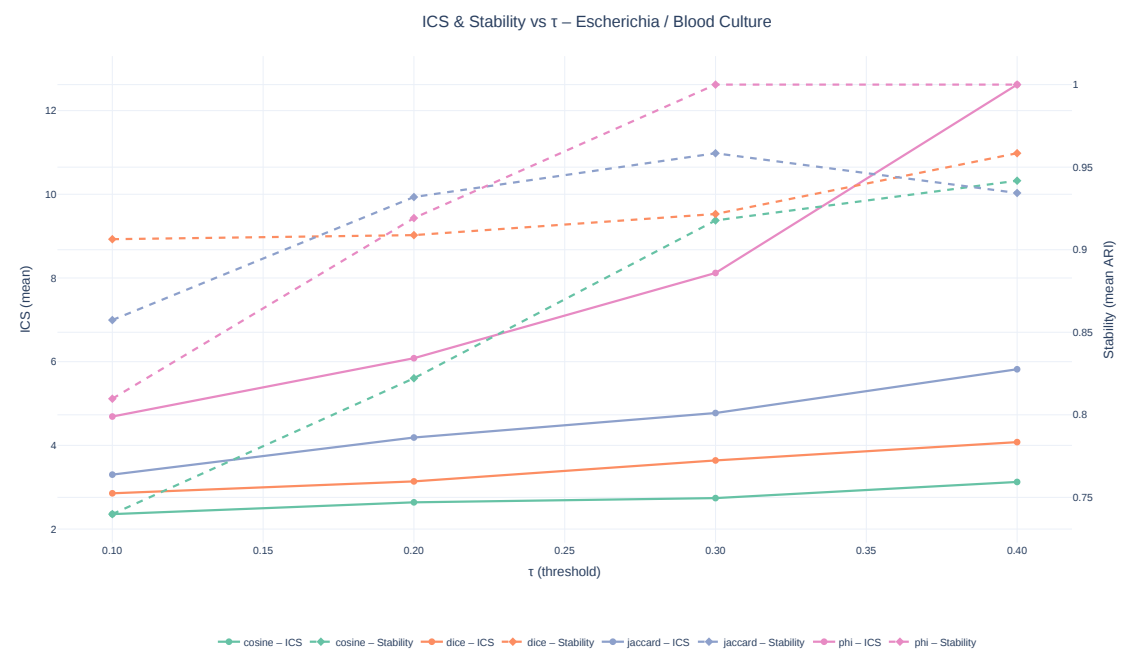

(A) *Escherichia*, blood culture

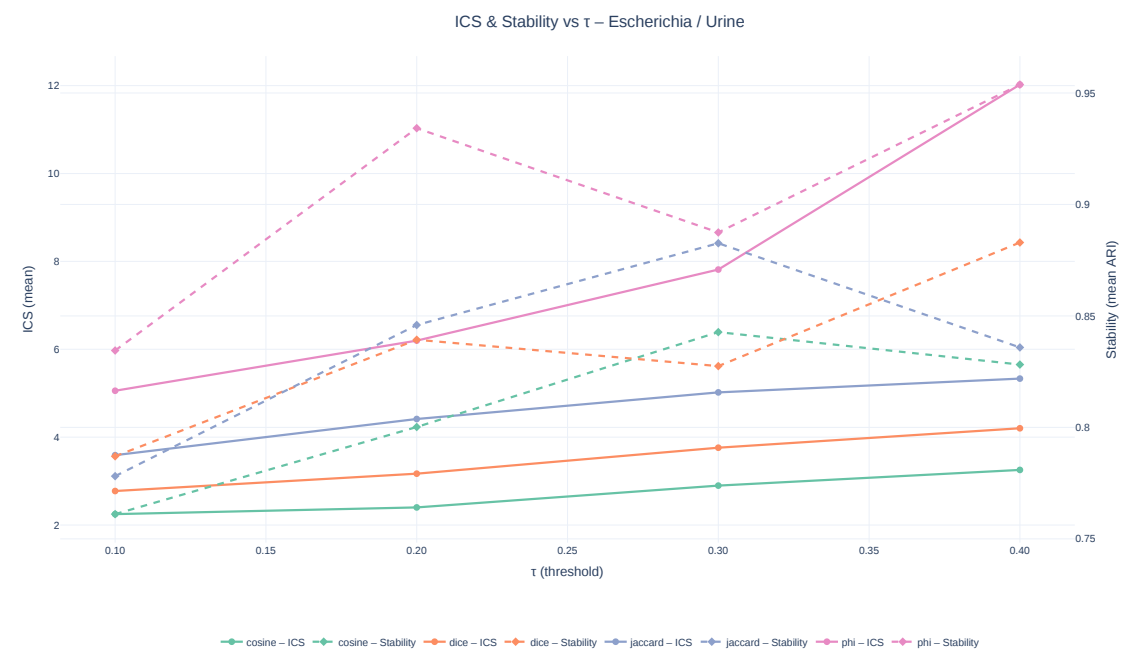

(B) *Escherichia*, urine

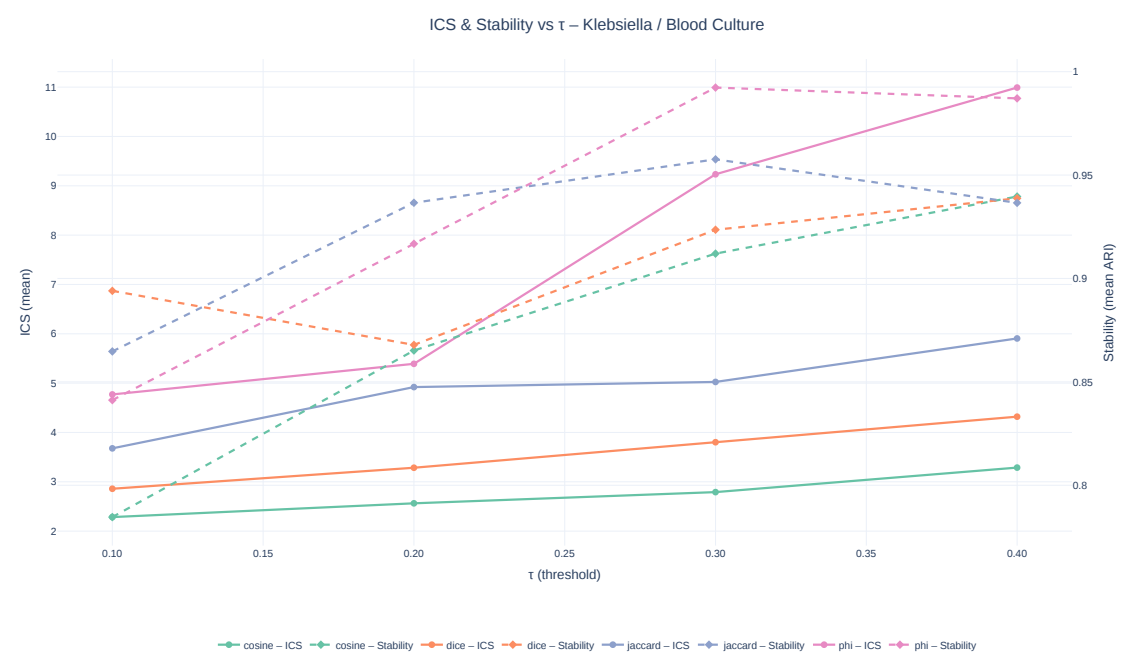

(C) *Klebsiella*, blood culture

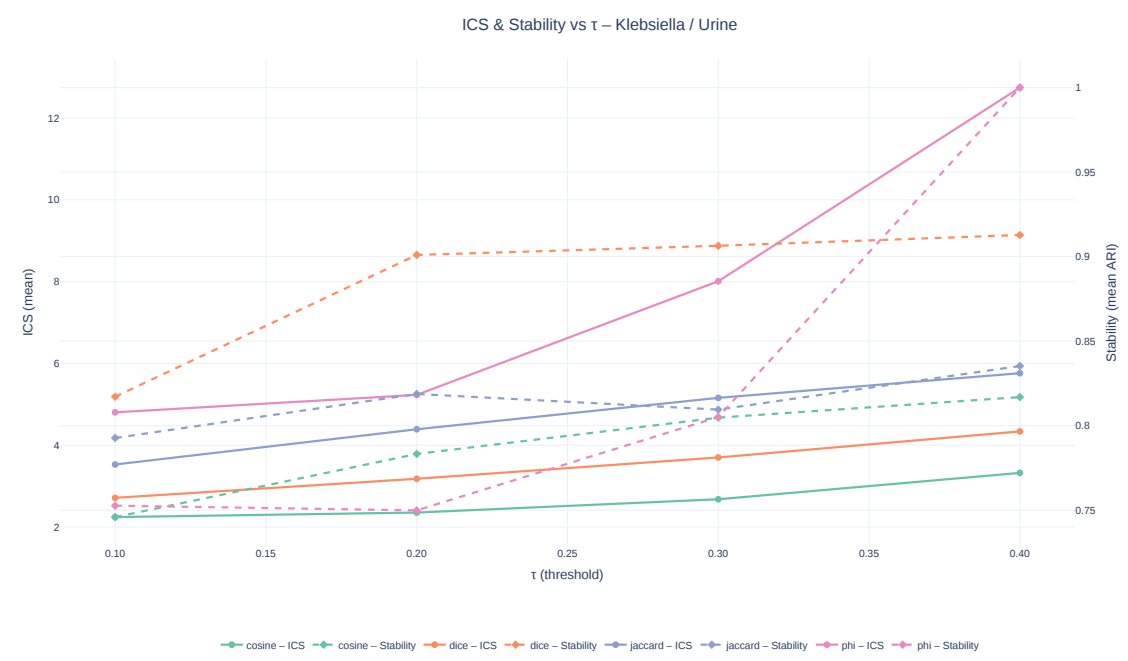

(D) *Klebsiella*, urine

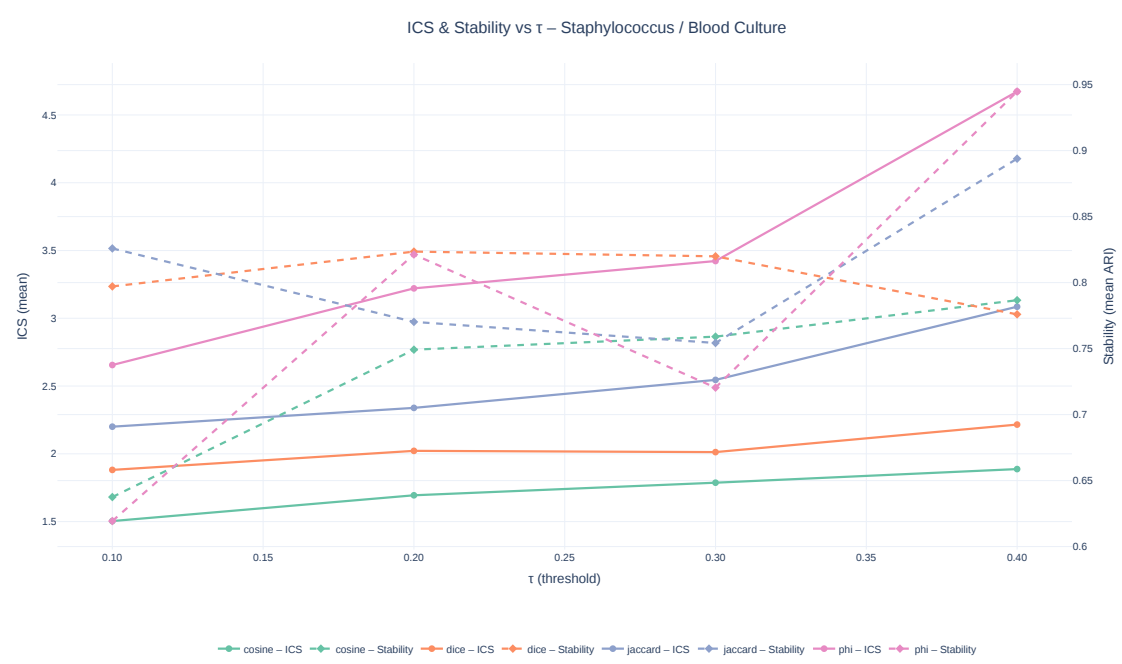

(E) *Staphylococcus*, blood culture

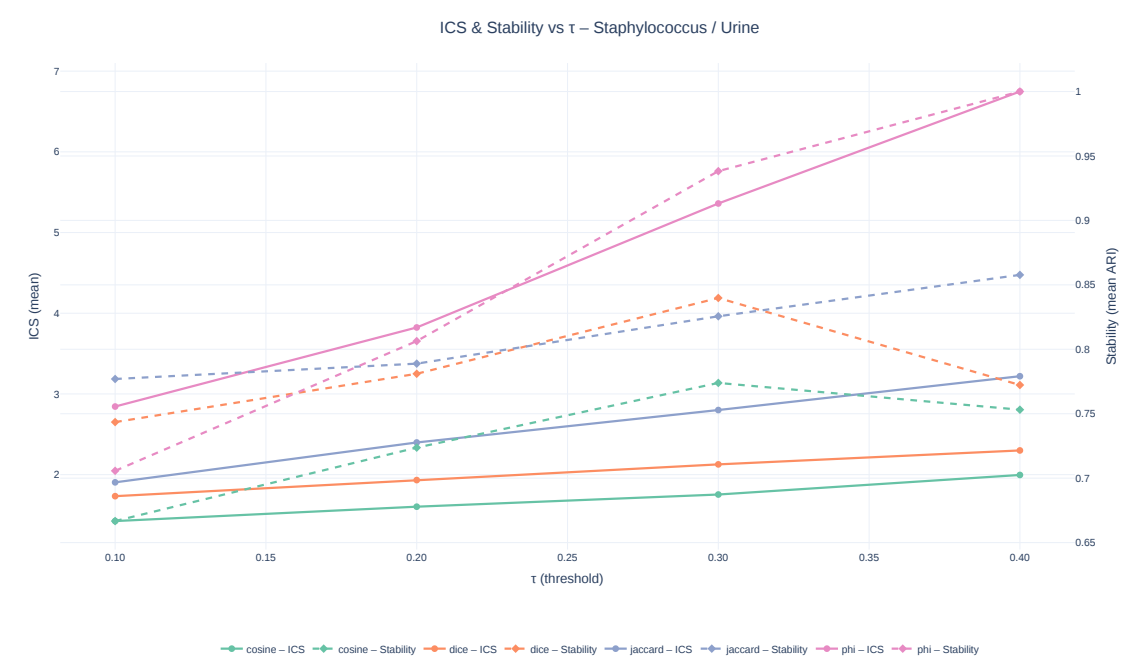

(F) *Staphylococcus*, urine

Figure S2: Stability–ICS performance landscapes across genera and specimen types. Each panel displays the joint behaviour of internal cluster separation (ICS score) and network stability across the similarity threshold and resolution parameter sweep. Panels (A–B) correspond to *Escherichia*, (C–D) to *Klebsiella*, and (E–F) to *Staphylococcus*, each shown for blood culture and urine specimens. Across all three genera, pronounced specimen-specific optimisation landscapes are observed, revealing distinct trade-offs between community cohesion and robustness. These results demonstrate that optimal co-testing network configurations are strongly dependent on both pathogen identity and specimen type, supporting stratified parameter tuning rather than a single global optimisation strategy.

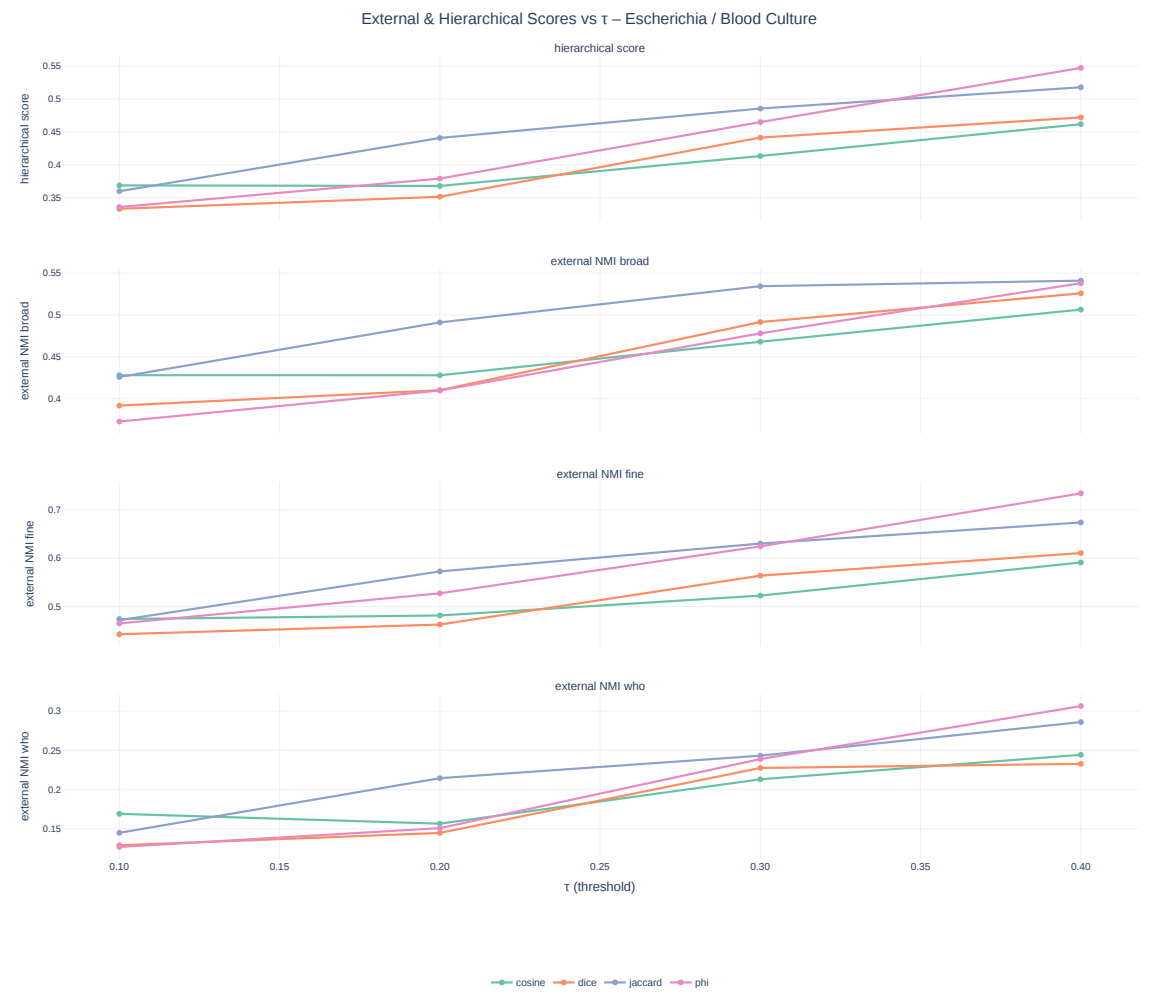

(A) *Escherichia*, blood culture

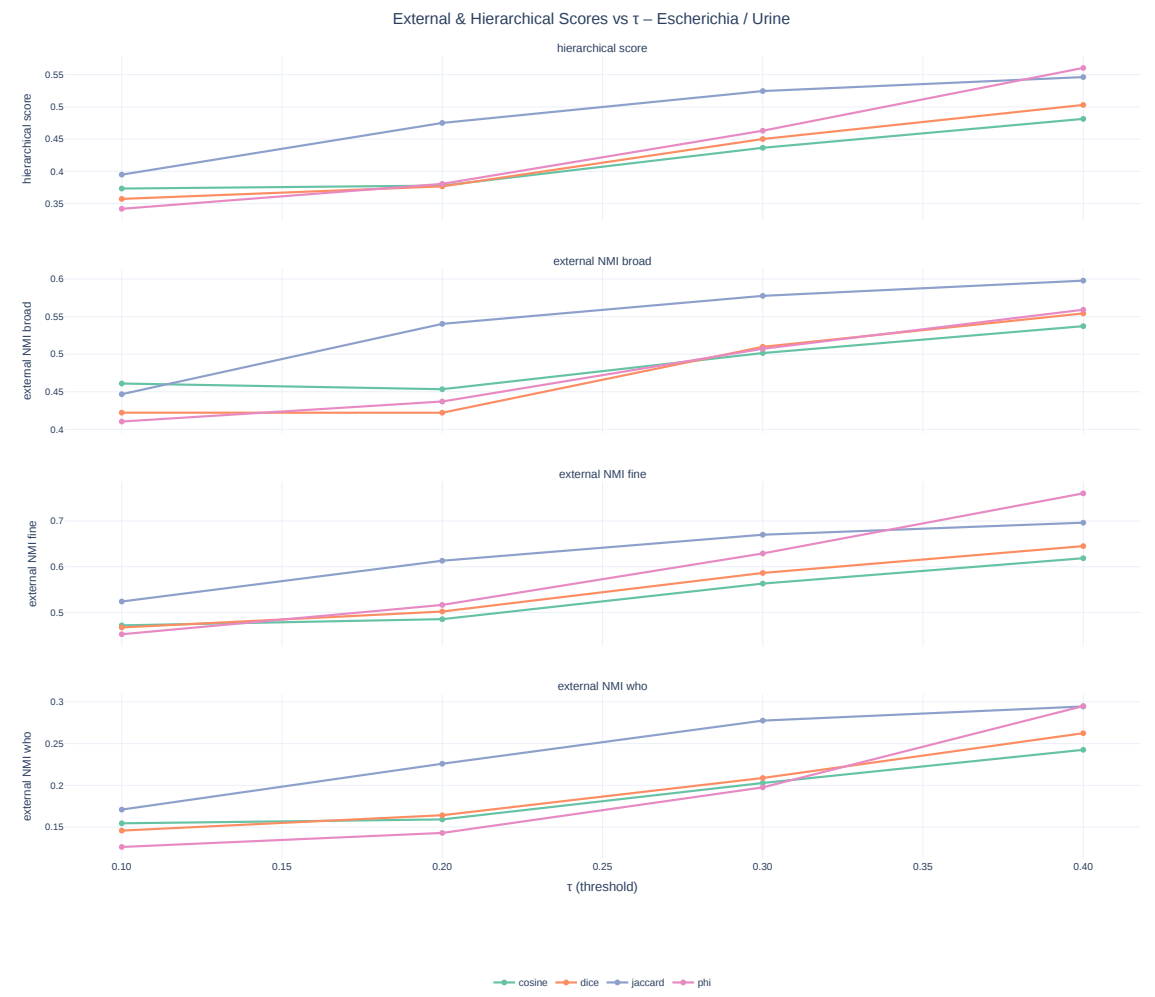

(B) *Escherichia*, urine

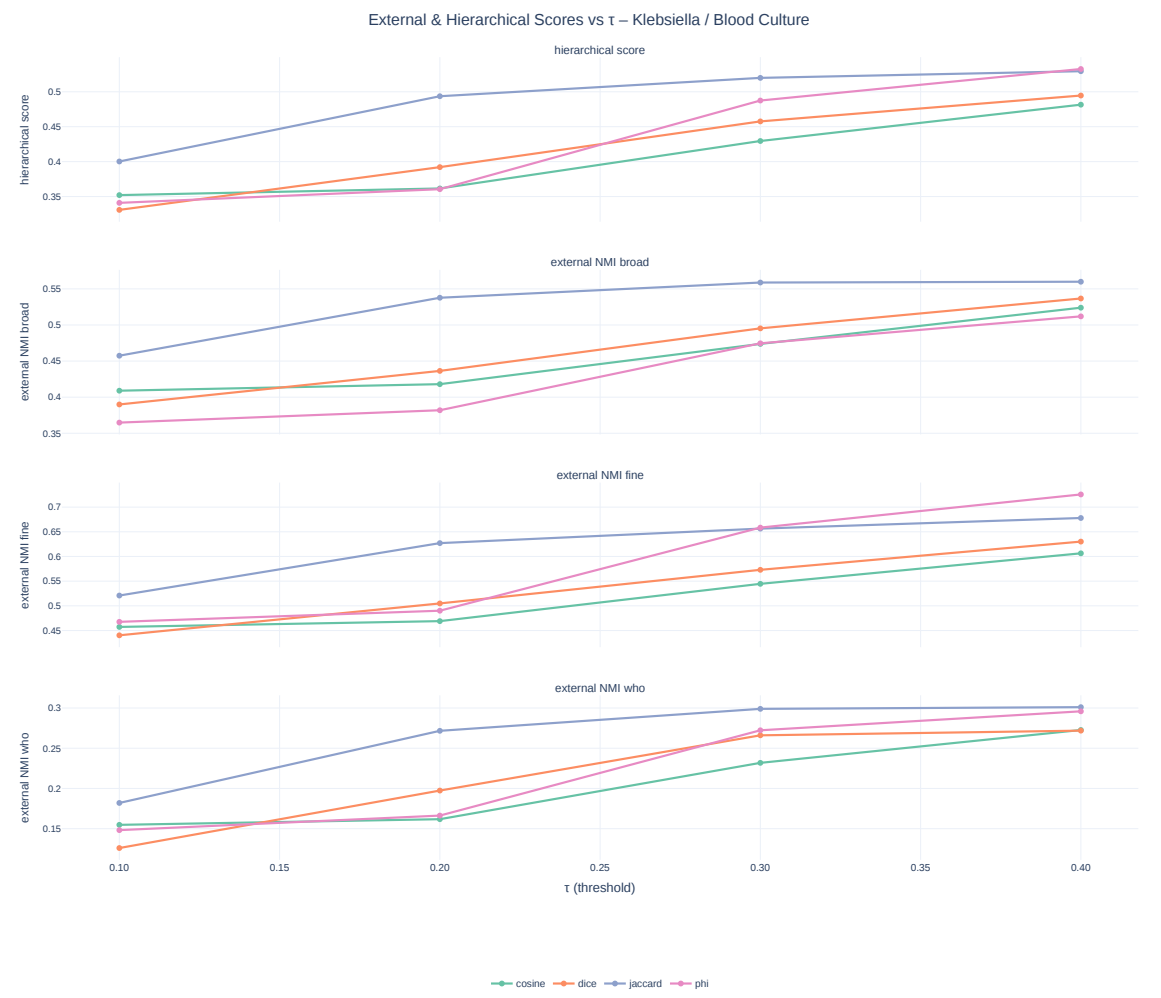

(C) *Klebsiella*, blood culture

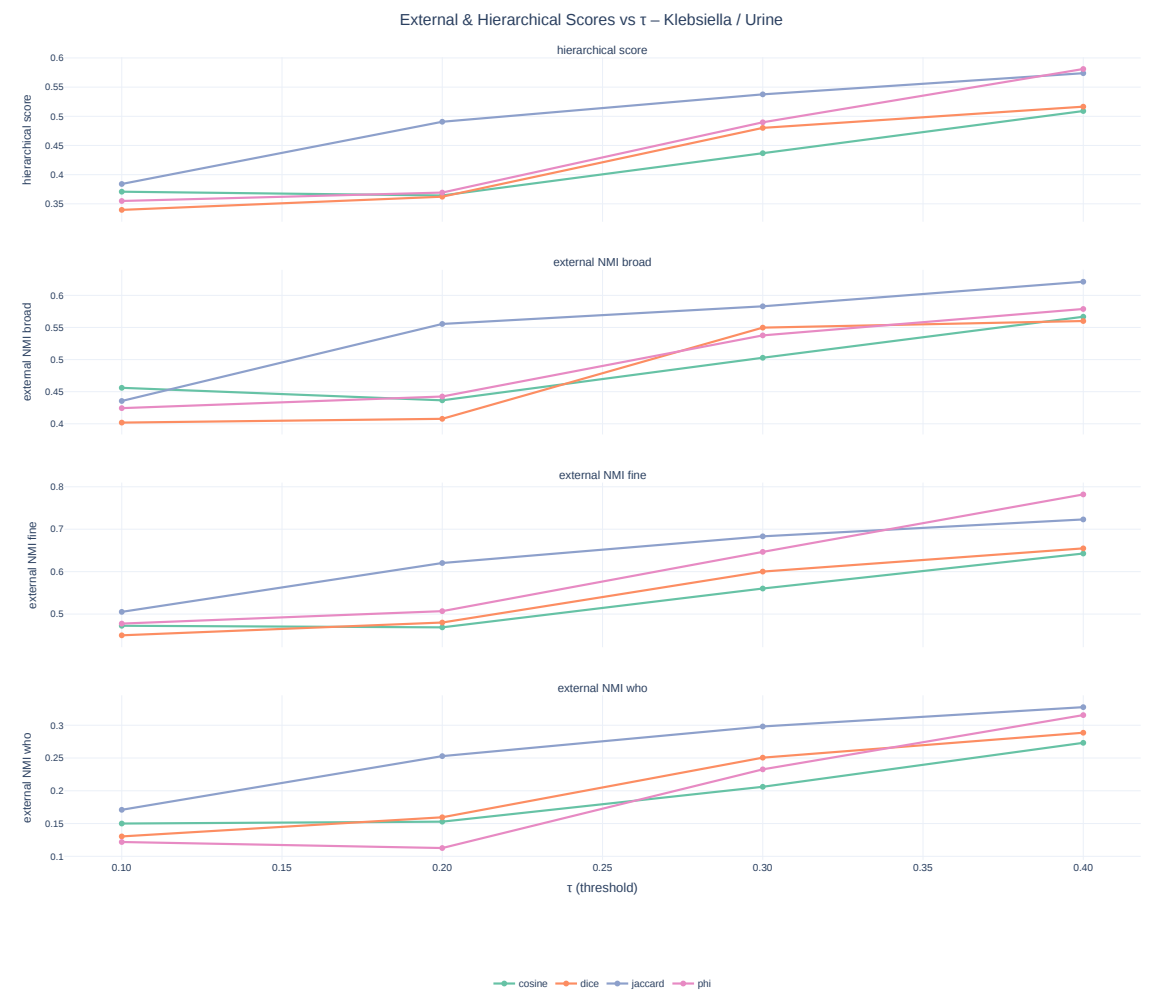

(D) *Klebsiella*, urine

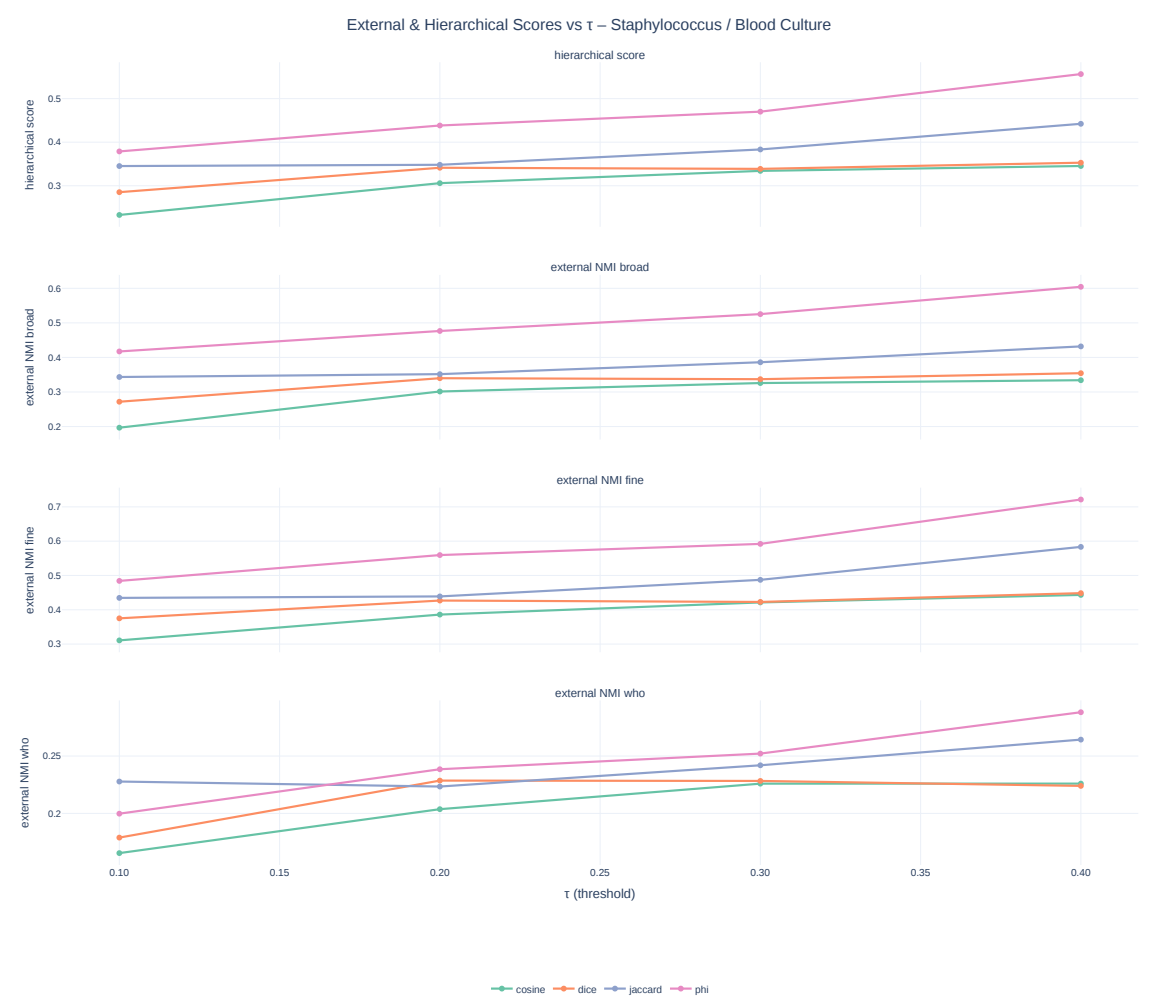

(E) *Staphylococcus*, blood culture

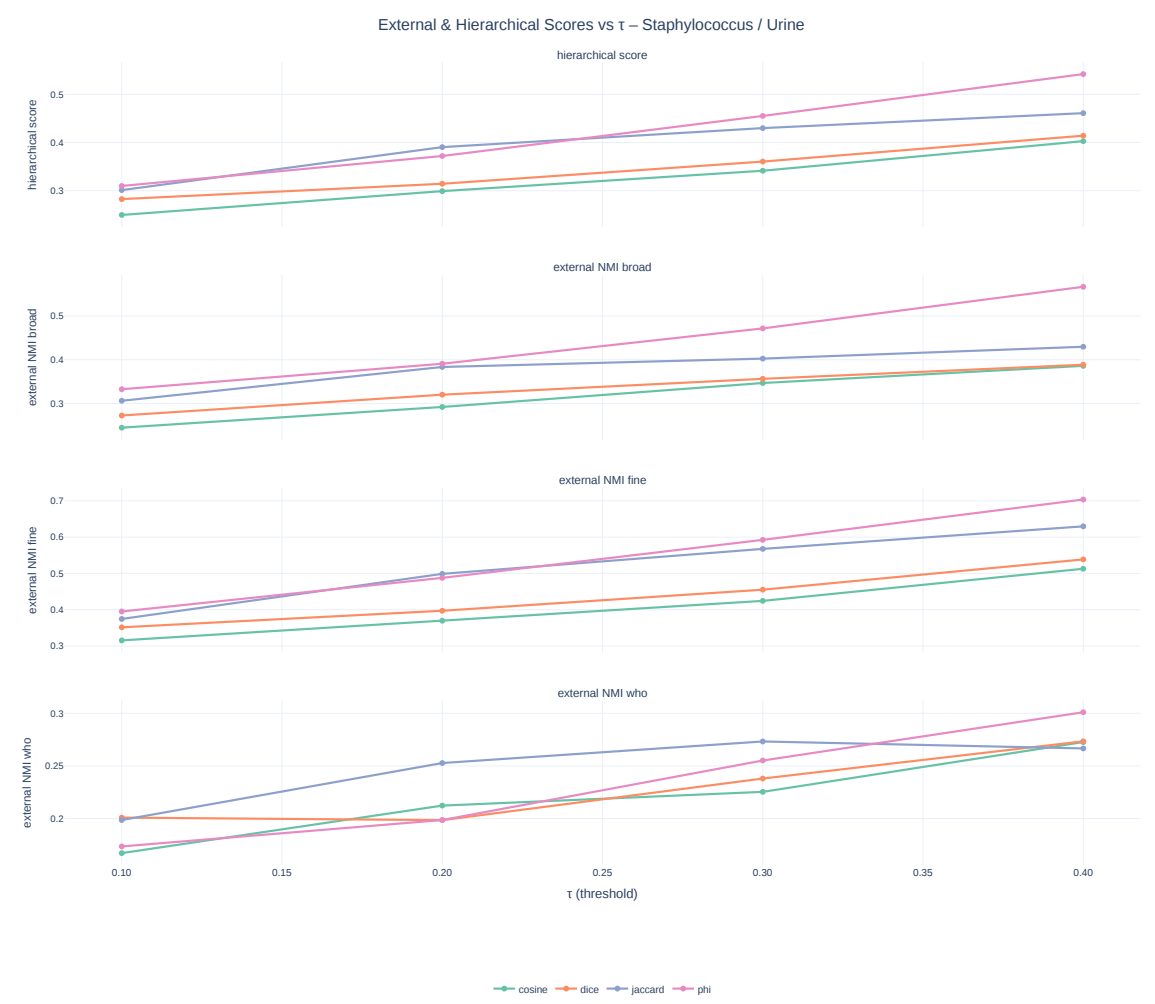

(F) *Staphylococcus*, urine

Figure S3: External validation performance across similarity parameter sweeps for three major genera. Each panel shows the behaviour of external agreement metrics (Adjusted Rand Index and Normalised Mutual Information) with respect to reference classifications across the similarity threshold and resolution parameter space. Panels (A–B) correspond to *Escherichia*, (C–D) to *Klebsiella*, and (E–F) to *Staphylococcus*, each stratified by blood culture and urine specimens. Across all genera, external agreement surfaces exhibit marked specimen-specific structure with distinct regions of optimal concordance. These patterns indicate that alignment with established phenotypic and stewardship-based classifications depends jointly on pathogen identity and specimen context, reinforcing the necessity of stratified optimisation in co-testing network inference.

Table S5: Antibiotics retained for analysis with ≥1% test coverage across all patient isolates. "Tested %" represents the proportion of isolates for which susceptibility testing was performed over the full study period.

Code	Antibiotic Name	Tested Count	Tested (%)
SXT	Co-Trimoxazol	11 961 072	92.07
CIP	Ciprofloxacin	10 895 506	83.87
MER	Meropenem	10 352 434	79.69
PIT	Piperacillin/Tazobactam	10 270 428	79.06
CAZ	Ceftazidim	9 233 667	71.08
CXM	Cefuroxim	9 134 555	70.32
AMP	Ampicillin	9 069 033	69.81
CTX	Cefotaxim	8 889 952	68.43
LEV	Levofloxacin	8 724 477	67.16
GEN	Gentamicin	8 238 198	63.42
AMS	Ampicillin/Sulbactam	8 066 141	62.09
AMC	Amoxicillin/clavulanic acid	7 564 396	58.23
IMP	Imipenem	7 521 182	57.9
PIP	Piperacillin	6 905 996	53.16
CRO	Ceftriaxon	5 847 101	45.01
CPO	Cefpodoxim	5 477 546	42.17
NFT	Nitrofurantoin	5 060 465	38.95
ERT	Ertapenem	5 020 813	38.65
TGC	Tigecyclin	4 219 512	32.48
CEP	Cefepim	3 885 658	29.91
TRP	Trimethoprim	3 720 674	28.64
MOX	Moxifloxacin	3 646 954	28.07
MCL	Mecillinam	3 244 785	24.98
CFA	Cefuroxim-Axetil	3 107 114	23.92
AMX	Amoxicillin	3 060 780	23.56
ERY	Erythromycin	2 884 109	22.2
VAN	Vancomycin	2 873 264	22.12
CLI	Clindamycin	2 851 504	21.95
LIZ	Linezolid	2 584 397	19.89
TET	Tetracyclin	2 577 116	19.84
TOB	Tobramycin	2 396 456	18.45
PEN	Penicillin	2 337 512	17.99
OXA	Oxacillin	2 109 266	16.24
TPL	Teicoplanin	1 942 462	14.95
CEZ	Cefazolin	1 921 005	14.79
CFI	Cefixim	1 897 773	14.61
RAM	Rifampicin	1 859 077	14.31
AMK	Amikacin	1 752 941	13.49
DOX	Doxycyclin	1 628 981	12.54
FUS	Fusidic acid	1 562 406	12.03
CEC	Cefaclor	1 427 220	10.99
DPT	Daptomycin	1 238 619	9.53

Continued on next page

Table S5 – continued from previous page

Code	Antibiotic Name	Tested Count	Tested (%)
AZT	Aztreonam	939 127	7.23
CEX	Cefalexin	800 483	6.16
CPP	Cefpodoxim-Proxetil	645 273	4.97
AZM	Azithromycin	627 906	4.83
CLR	Clarithromycin	597 442	4.6
CMP	Chloramphenicol	429 998	3.31
FLU	Flucloxacillin	343 183	2.64
NOR	Norfloxacin	335 375	2.58
COX	Cefoxitin	314 197	2.42
COL	Colistin	311 101	2.39
OFX	Ofloxacin	225 705	1.74
CFR	Cefadroxil	221 816	1.71
ROX	Roxithromycin	190 859	1.47
MZL	Mezlocillin	187 944	1.45
CZA	Ceftazidim/Avibactam	184 263	1.42

Table S6: Antibiotics with < 1% test coverage that were excluded from analysis ("Dropped"). "Tested %" is the fraction of isolates in which the agent was tested, computed over the full study period.

Antibiotic	Tested Count	Tested (%)	Coverage category
SPF - Sparfloxacin	1	0.0	Dropped
SAZ - Sulphamethoxazol	1	0.0	Dropped
FID - Fidaxomicin	1	0.0	Dropped
LOM - Lomefloxacin	1	0.0	Dropped
TFX - Trovafloxacin	2	0.0	Dropped
ETH - Ethionamid	2	0.0	Dropped
CMX - Cefmenoxim	2	0.0	Dropped
CEQ - Cefqinom	3	0.0	Dropped
CXC - Cefotaxim/clavulanic acid	4	0.0	Dropped
CAR - Carbenicillin	4	0.0	Dropped
CMD - Cefamandol	5	0.0	Dropped
MZS - Sulbactam/Mezlocillin	6	0.0	Dropped
ORI - Oritavancin	7	0.0	Dropped
SFX - Sulfamethizol	7	0.0	Dropped
CPZ - Cefoperazon	8	0.0	Dropped
CPC - Cefpodoxim/clavulanic acid	10	0.0	Dropped
ENO - Enoxacin	22	0.0	Dropped
CMT - Cefmetazol	22	0.0	Dropped
CED - Cefradin	22	0.0	Dropped
PIM - Pipemidic acid	22	0.0	Dropped
AZL - Azlocillin	23	0.0	Dropped
TOS - Tosufloxacin	29	0.0	Dropped
DEL - Delafloxacin	32	0.0	Dropped

Continued on next page

Table S6 – continued from previous page

Antibiotic	Tested Count	Tested (%)	Coverage category
CSE - Cycloserin	49	0.0	Dropped
MOL - Moxalactam	63	0.0	Dropped
NOV - Novobiocin	67	0.0	Dropped
PAS - p-Aminosalicylic acid	104	0.0	Dropped
CFS - Cefsulodin	152	0.0	Dropped
CPR - Capreomycin	156	0.0	Dropped
DAL - Dalbavancin	176	0.0	Dropped
MAR - Marbofloxacin	190	0.0	Dropped
PTH - Protionamid	239	0.0	Dropped
CTT - Cefotetan	333	0.0	Dropped
KET - Ketoconazol	427	0.0	Dropped
SPC - Spectinomycin	438	0.0	Dropped
CZC - Ceftazidim/clavulanic acid	537	0.0	Dropped
PIV - Pivampicillin	663	0.0	Dropped
CFL - Ceftiofur	767	0.0	Dropped
IVC - Isavuconazol	884	0.01	Dropped
ISO - Isooxazolylpenicillin	1255	0.01	Dropped
CIB - Ceftibuten	1562	0.01	Dropped
MTR - Metronidazol	1040	0.01	Dropped
NAL - Nalidixic acid	2120	0.01	Dropped
MET - Methicillin	2634	0.02	Dropped
NET - Netilmicin	2834	0.02	Dropped
STR - Streptomycin	3286	0.02	Dropped
TEL - Telithromycin	3637	0.02	Dropped
ECO - Econazol	4570	0.03	Dropped
CTR - Clotrimazol	4596	0.03	Dropped
SONST - Other antibiotic	4646	0.03	Dropped
MCZ - Miconazol	4753	0.03	Dropped
LIN - Lincomycin	5676	0.03	Dropped
MNO - Minocyclin	5957	0.03	Dropped
INH - Isoniazid	6383	0.04	Dropped
PZA - Pyrazinamid	6532	0.04	Dropped
JOS - Josamycin	6686	0.04	Dropped
EMB - Ethambutol	6746	0.04	Dropped
NYS - Nystatin	6823	0.04	Dropped
BTC - Bacitracin	7575	0.04	Dropped
NEO - Neomycin	8926	0.05	Dropped
PRI - Pristinamycin	10391	0.06	Dropped
CZX - Ceftizoxim	10602	0.06	Dropped
CAP - Cefapirin	10653	0.06	Dropped
TMO - Temocillin	10782	0.06	Dropped
KAN - Kanamycin	12861	0.07	Dropped
CCO - Cefiderocol	12913	0.07	Dropped
ITR - Itraconazol	17248	0.1	Dropped

Continued on next page

Table S6 – continued from previous page

Antibiotic	Tested Count	Tested (%)	Coverage category
PEF - Perfloxacin	17428	0.1	Dropped
CTL - Ceftarolin	20543	0.12	Dropped
POS - Posaconazol	22854	0.13	Dropped
CTN - Cefalotin	29197	0.17	Dropped
TIC - Ticarcillin	36261	0.21	Dropped
RIB - Rifabutin	43819	0.25	Dropped
PIC - Pivmecillinam	44247	0.26	Dropped
DOR - Doripenem	47626	0.27	Dropped
POL - Polymyxin	49903	0.29	Dropped
ANI - Anidulafungin	55225	0.32	Dropped
5FC - 5-Fluorcytosin	71522	0.41	Dropped
TCC - Ticarcillin/clavulanic acid	80715	0.47	Dropped
MIC - Micafungin	81234	0.47	Dropped
PIS - Piperacillin/Sulbactam	91725	0.53	Dropped
SHL - Streptomycin 1000 (high level)	107546	0.62	Dropped
LOR - Loracarbef	109028	0.63	Dropped
ESBL - Extended ß-Lactamase	112527	0.65	Dropped
CTM - Cefotiam	136306	0.79	Dropped
VOR - Voriconazol	139429	0.8	Dropped
CZT - Ceftolozan/Tazobactam	142987	0.82	Dropped
CAS - Caspofungin	143440	0.83	Dropped

Table S7: Antibiotics never tested in the dataset. These agents were present in the reference list but had no recorded susceptibility testing during the study period.

**Code**	**Antibiotic Name**
SLD	Sulfadiazin
CLN	Clinafloxacin
APL	Apalcillin
BAC	Bacampicillin
CDT	Cefditoren
CFD	Cefdinir
CFT	Cefetamet
CIN	Cinoxacin
CLO	Cefaloridin
CLX	Cloxiquin
CNI	Cefonicid
COM	Cotrimazol
CPI	Cefpirom
CPY	Cefprozil
CRI	Cefatrizin
CTE	Chlortetracyclin
CZD	Cefazedon
DAN	Danofloxacin

Continued on next page

Code	Antibiotic Name
DIC	Dicloxacillin
DIR	Dirithromycin
FLE	Fleroxacillin
FRA	Framycetin
GAT	Gatifloxacin
GEM	Gemifloxacin
GRE	Grepafloxacin
ISE	Isepamicin
KHL	Kanamycin (high level)
LAM	Lampren
LMO	Latamofex
MID	Midekamycin
OLE	Oleandomycin
PAN	Panipenem
PAR	Paromycin
ROC	Rocephin
ROS	Rosoxacin
SIS	Sisomycin
SLN	Sulfalen
SOX	Sulfisoxazol
SPM	Spiramycin
SUL	Sulbactam
TLV	Telavancin
BPR	Ceftobiprol
TZD	Tedizolid
OMA	Omadacyclin
PLA	Plazomicin
LEF	Lefamulin

Table S8: Distribution of bacterial genera by Gram-stain and specimen type.

Gram-stain	Bacterium	Urine	Wound	Swab	Others	Blood culture	Respiratory	Stool	Punctate	Urogenital swab	Exclusion	Total
Gram-positive (n=3279756)												
	Staphylococcus	125181 (5.9)	701092 (32.8)	754410 (35.3)	121261 (5.7)	148224 (6.9)	142190 (6.7)	5207 (0.2)	94512 (4.4)	45925 (2.1)	11 (0.0)	2138013 (16.5)
	Streptococcus	160064 (24.3)	94997 (14.4)	183590 (27.9)	6827 (1.0)	33149 (5.0)	24787 (3.8)	211 (0.0)	11870 (1.8)	143101 (21.7)	1 (0.0)	658597 (5.1)
	Enterococcus	178293 (36.9)	84238 (17.4)	87760 (18.2)	51606 (10.7)	33366 (6.9)	7203 (1.5)	6095 (1.3)	32476 (6.7)	2102 (0.4)	7 (0.0)	483146 (3.7)
Gram-negative (n=9702521)												
	Escherichia	3360248 (71.1)	329422 (7.0)	299654 (6.3)	123366 (2.6)	238870 (5.1)	110102 (2.3)	12260 (0.3)	64678 (1.4)	190230 (4.0)	37 (0.0)	4728867 (36.4)
	Klebsiella	831833 (52.4)	178286 (11.2)	212722 (13.4)	65899 (4.2)	75632 (4.8)	145162 (9.2)	3791 (0.2)	29885 (1.9)	42758 (2.7)	5 (0.0)	1585973 (12.2)

Continued on next page

Table S8 – continued from previous page

Gram-stain	Bacterium	Urine	Wound	Swab	Others	Blood culture	Respiratory	Stool	Punctate	Urogenital swab	Exclusion	Total
	Pseudomonas	318726 (32.7)	221787 (22.8)	200262 (20.6)	49448 (5.1)	21986 (2.3)	130008 (13.3)	4221 (0.4)	20149 (2.1)	7861 (0.8)	14 (0.0)	974462 (7.5)
	Proteus	525276 (56.6)	208907 (22.5)	80668 (8.7)	14234 (1.5)	28488 (3.1)	33713 (3.6)	250 (0.0)	17552 (1.9)	19321 (2.1)	6 (0.0)	928415 (7.1)
	Enterobacter	153207 (31.1)	120708 (24.5)	101871 (20.7)	25631 (5.2)	19382 (3.9)	46927 (9.5)	1282 (0.3)	17820 (3.6)	5256 (1.1)	4 (0.0)	492088 (3.8)
	Citrobacter	178638 (51.3)	53743 (15.4)	53338 (15.3)	14115 (4.0)	8800 (2.5)	22942 (6.6)	616 (0.2)	8368 (2.4)	7971 (2.3)	0 (0.0)	348531 (2.7)
	Serratia	50538 (24.6)	48558 (23.6)	37886 (18.4)	6873 (3.3)	9060 (4.4)	45743 (22.2)	76 (0.0)	5413 (2.6)	1507 (0.7)	1 (0.0)	205655 (1.6)
	Morganella	90376 (54.1)	39884 (23.9)	15907 (9.5)	3968 (2.4)	3631 (2.2)	4859 (2.9)	80 (0.0)	4104 (2.5)	4194 (2.5)	0 (0.0)	167003 (1.3)
	Haemophilus	58 (0.0)	1388 (1.0)	88479 (66.4)	1098 (0.8)	2314 (1.7)	38178 (28.6)	2 (0.0)	464 (0.3)	1305 (1.0)	0 (0.0)	133286 (1.0)
	Acinetobacter	14631 (17.0)	23933 (27.8)	27155 (31.5)	8914 (10.3)	1624 (1.9)	7253 (8.4)	104 (0.1)	1364 (1.6)	1209 (1.4)	0 (0.0)	86187 (0.7)

Continued on next page

Table S8 – continued from previous page

Gram-stain	Bacterium	Urine	Wound	Swab	Others	Blood culture	Respiratory	Stool	Punctate	Urogenital swab	Exclusion	Total
	Providencia	21357 (50.4)	13178 (31.1)	3972 (9.4)	572 (1.3)	1114 (2.6)	1156 (2.7)	11 (0.0)	792 (1.9)	240 (0.6)	0 (0.0)	42392 (0.3)
	Neisseria	203 (4.1)	82 (1.7)	724 (14.6)	58 (1.2)	11 (0.2)	0 (0.0)	0 (0.0)	29 (0.6)	3841 (77.6)	0 (0.0)	4948 (0.0)
	Salmonella	346 (7.4)	77 (1.6)	27 (0.6)	18 (0.4)	464 (9.9)	12 (0.3)	3702 (79.1)	31 (0.7)	3 (0.1)	0 (0.0)	4680 (0.0)
	Shigella	4 (15.4)	0 (0.0)	0 (0.0)	0 (0.0)	0 (0.0)	0 (0.0)	22 (84.6)	0 (0.0)	0 (0.0)	0 (0.0)	26 (0.0)
	Obesumbacterium	2 (25.0)	0 (0.0)	1 (12.5)	0 (0.0)	0 (0.0)	2 (25.0)	0 (0.0)	2 (25.0)	1 (12.5)	0 (0.0)	8 (0.0)
Miscellaneous (n=8380)												
	Mycobacterium	111 (1.3)	112 (1.3)	58 (0.7)	756 (9.0)	14 (0.2)	5994 (71.5)	12 (0.1)	1259 (15.0)	64 (0.8)	0 (0.0)	8380 (0.1)

Table S9: Age-group and sex distribution of isolates for the most frequent bacterial genera.

Bacterium	< 15	15–29	30–44	45–59	60–74	75–89	90+	Woman	Man	Others	Total
Escherichia	264672 (2.8)	279061 (3.0)	377506 (4.0)	606739 (6.4)	1200310 (12.7)	1729263 (18.3)	268622 (2.8)	2937402 (31.1)	1194842 (12.6)	596623 (6.3)	9455040 (36.4)
Staphylococcus	193797 (4.5)	161538 (3.8)	194779 (4.6)	324486 (7.6)	540147 (12.6)	633172 (14.8)	88906 (2.1)	795298 (18.6)	1044725 (24.4)	297990 (7.0)	4274838 (16.5)
Klebsiella	103997 (3.3)	55311 (1.7)	90140 (2.8)	198192 (6.3)	466177 (14.7)	597796 (18.9)	73426 (2.3)	700034 (22.1)	677237 (21.4)	208702 (6.6)	3171012 (12.2)
Pseudomonas	33354 (1.7)	28088 (1.4)	47896 (2.5)	124607 (6.4)	302886 (15.5)	387226 (19.9)	49871 (2.6)	330692 (17.0)	506130 (26.0)	137640 (7.1)	1948390 (7.5)
Proteus	27646 (1.5)	24203 (1.3)	36426 (2.0)	101753 (5.5)	260116 (14.0)	412533 (22.2)	65294 (3.5)	416595 (22.4)	383735 (20.7)	128085 (6.9)	1856386 (7.1)
Streptococcus	87540 (6.6)	91024 (6.9)	144640 (11.0)	110466 (8.4)	125146 (9.5)	89360 (6.8)	10097 (0.8)	369811 (28.1)	204004 (15.5)	84782 (6.4)	1316870 (5.1)
Enterobacter	48240 (4.9)	13461 (1.4)	24701 (2.5)	64970 (6.6)	149977 (15.2)	170774 (17.4)	19644 (2.0)	182472 (18.5)	244611 (24.9)	65005 (6.6)	983855 (3.8)
Enterococcus	5841 (0.6)	6642 (0.7)	18583 (1.9)	64722 (6.7)	168302 (17.4)	198009 (20.5)	20760 (2.1)	193979 (20.1)	218644 (22.6)	70523 (7.3)	966005 (3.7)

Continued on next page

Table S9 – continued from previous page

Bacterium	< 15	15–29	30–44	45–59	60–74	75–89	90+	Woman	Man	Others	Total
Citrobacter	16587 (2.4)	11721 (1.7)	19457 (2.8)	44222 (6.3)	101569 (14.6)	138362 (19.9)	16422 (2.4)	131876 (18.9)	172717 (24.8)	43938 (6.3)	696871 (2.7)
Serratia	11563 (2.8)	6159 (1.5)	10576 (2.6)	28579 (7.0)	68674 (16.7)	73598 (17.9)	6353 (1.5)	61025 (14.8)	117358 (28.5)	27272 (6.6)	411157 (1.6)
Morganella	3974 (1.2)	2857 (0.9)	6742 (2.0)	20115 (6.0)	51272 (15.4)	71346 (21.4)	10605 (3.2)	66068 (19.8)	78007 (23.4)	22928 (6.9)	333914 (1.3)
Haemophilus	41969 (15.7)	18265 (6.9)	22697 (8.5)	17471 (6.6)	22353 (8.4)	9844 (3.7)	628 (0.2)	57819 (21.7)	55158 (20.7)	20309 (7.6)	266513 (1.0)
Acinetobacter	9527 (5.5)	3812 (2.2)	6305 (3.7)	12345 (7.2)	24046 (14.0)	26671 (15.5)	3440 (2.0)	29215 (17.0)	41516 (24.1)	15456 (9.0)	172333 (0.7)
Providencia	453 (0.5)	810 (1.0)	1673 (2.0)	5163 (6.1)	12762 (15.1)	18468 (21.8)	3045 (3.6)	12979 (15.3)	23652 (27.9)	5761 (6.8)	84766 (0.3)
Mycobacterium	213 (1.3)	1922 (11.5)	1866 (11.1)	1592 (9.5)	1513 (9.0)	1187 (7.1)	70 (0.4)	2480 (14.8)	4337 (25.9)	1563 (9.3)	16743 (0.1)
Neisseria	33 (0.3)	2017 (20.4)	1681 (17.0)	958 (9.7)	229 (2.3)	27 (0.3)	1 (0.0)	642 (6.5)	3627 (36.7)	679 (6.9)	9894 (0.0)

Continued on next page

Table S9 – continued from previous page

Bacterium	< 15	15–29	30–44	45–59	60–74	75–89	90+	Woman	Man	Others	Total
Salmonella	1374 (14.7)	808 (8.6)	423 (4.5)	518 (5.5)	755 (8.1)	734 (7.8)	67 (0.7)	1916 (20.5)	2394 (25.6)	370 (4.0)	9359 (0.0)
Shigella	1 (1.9)	7 (13.5)	4 (7.7)	7 (13.5)	5 (9.6)	1 (1.9)	1 (1.9)	10 (19.2)	9 (17.3)	7 (13.5)	52 (0.0)
Obesumbacterium	0 (0.0)	0 (0.0)	1 (6.2)	0 (0.0)	1 (6.2)	6 (37.5)	0 (0.0)	4 (25.0)	4 (25.0)	0 (0.0)	16 (0.0)

Table S10: Total number of antimicrobial susceptibility tests ($\sum T$) for major Gram-negative pathogens by agent.

Antimicrobial	*Pseudomonas* spp. $\sum T$	*Klebsiella* spp. $\sum T$	*Proteus* spp. $\sum T$	*E. coli* $\sum T$	*Enterobacter* spp. $\sum T$	*Salmonella typhi* $\sum T$	Total $\sum T$
Ciprofloxacin	972724	1580380	925401	4702919	490408	164	8671996
Meropenem	954408	1520526	884582	4439076	474707	171	8273470
Piperacillin/Tazobactam	943408	1508946	882378	4398785	471392	169	8205078
Co-Trimoxazol	488786	1578755	925091	4707283	488956	171	8189042
Ceftazidim	966576	1501432	874322	4364934	470529	165	8177958
Cefotaxim	358285	1412288	818590	4141626	438226	154	7169169

Continued on next page

Table S10 – continued from previous page

Antimicrobial	*Pseudomonas* spp. $\sum T$	*Klebsiella* spp. $\sum T$	*Proteus* spp. $\sum T$	*E. coli* $\sum T$	*Enterobacter* spp. $\sum T$	*Salmonella typhi* $\sum T$	Total $\sum T$
Cefuroxim	231437	1362107	777424	4116673	211882	59	6699582
Ampicillin	226759	1333996	771619	3880237	395019	154	6607784
Piperacillin	837871	1043320	582562	2914128	326798	118	5704797
Gentamicin	355108	1129951	646556	2797658	401309	156	5330738
Ampicillin/Sulbactam	251591	1119262	652628	2933748	346510	162	5303901
Amoxicillin/clavulanic acid	234863	952186	571057	3276709	256787	64	5291666
Levofloxacin	358660	1002566	586241	3021968	304962	82	5274479
Imipenem	930810	1003486	455427	2326276	375132	162	5091293
Cefpodoxim	48453	862149	525430	2892324	236464	60	4564880
Nitrofurantoin	98569	415656	302059	3433771	95968	20	4346043
Ertapenem	116398	892516	500771	2534429	269973	100	4314187

Continued on next page

Table S10 – continued from previous page

Antimicrobial	*Pseudomonas* spp. $\sum T$	*Klebsiella* spp. $\sum T$	*Proteus* spp. $\sum T$	*E. coli* $\sum T$	*Enterobacter* spp. $\sum T$	*Salmonella typhi* $\sum T$	Total $\sum T$
Ceftriaxon	207077	892365	499903	2431933	279598	126	4311002
Cefepim	838723	502501	297483	1660270	141087	33	3440097
Trimethoprim	83136	554333	339365	2307127	115094	17	3399072
Mecillinam	53792	224048	122586	2607766	47318	7	3055517
Cefuroxim-Axetil	25162	478895	285158	1815392	57034	18	2661659
Tigecyclin	240746	168515	167289	1521778	62561	22	2160911
Amoxicillin	49518	408302	241831	1278525	118092	29	2096297
Tobramycin	841472	220686	113438	537824	78005	27	1791452
Moxifloxacin	93191	389655	180876	875633	144691	34	1684080
Amikacin	712018	182066	91555	476161	61972	18	1523790
Cefixim	37120	283769	174975	906567	76291	17	1478739

Continued on next page

Table S10 – continued from previous page

Antimicrobial	*Pseudomonas* spp. $\sum T$	*Klebsiella* spp. $\sum T$	*Proteus* spp. $\sum T$	*E. coli* $\sum T$	*Enterobacter* spp. $\sum T$	*Salmonella typhi* $\sum T$	Total $\sum T$
Aztreonam	552952	72259	37958	201111	22916	7	887203
Cefpodoxim-Proxetil	6192	98809	60186	350028	25239	12	540466
Cefalexin	12307	82686	56495	360548	17765	1	529802
Tetracyclin	72470	77644	72295	234841	27751	18	485019
Cefazolin	72624	83904	37360	202939	37096	17	433940
Doxycyclin	33793	85615	57143	225645	30609	10	432815
Cefaclor	40005	70982	44090	231915	23471	5	410468
Norfloxacin	47485	54697	28351	147683	18481	7	296704
Chloramphenicol	13095	54051	30278	166391	13473	3	277291
Colistin	103172	36179	28747	95451	11243	2	274794
Ofloxacin	6293	29229	15053	114340	7845	1	172761

Continued on next page

Table S10 – continued from previous page

Antimicrobial	*Pseudomonas* spp. $\sum T$	*Klebsiella* spp. $\sum T$	*Proteus* spp. $\sum T$	*E. coli* $\sum T$	*Enterobacter* spp. $\sum T$	*Salmonella typhi* $\sum T$	Total $\sum T$
Ceftazidim/Avibactam	50508	33264	12718	57050	12961	9	166510
Cefadroxil	4823	18508	11062	105461	3461	0	143315
Mezlocillin	9375	22694	12388	51884	8573	4	104918
Vancomycin	12045	21774	13647	47437	8619	2	103524
Erythromycin	11150	15769	12150	30664	7189	2	76924
Clindamycin	11377	15493	12087	29395	7383	1	75736
Oxacillin	9725	13667	10798	25727	6227	1	66145
Penicillin	6758	10944	7074	22509	4663	0	51948
Cefoxitin	6410	4662	2397	14362	4235	0	32066
Linezolid	4346	4976	4976	7921	2570	0	24789
Teicoplanin	2134	4291	2310	9672	1793	0	20200

Continued on next page

Table S10 – continued from previous page

Antimicrobial	*Pseudomonas* spp. $\sum T$	*Klebsiella* spp. $\sum T$	*Proteus* spp. $\sum T$	*E. coli* $\sum T$	*Enterobacter* spp. $\sum T$	*Salmonella typhi* $\sum T$	Total $\sum T$
Clarithromycin	1166	1476	786	2944	538	0	6910
Rifampicin	1164	1399	849	2239	717	0	6368
Fusidic acid	780	605	497	809	382	0	3073
Flucloxacillin	321	427	272	645	261	0	1926
Daptomycin	355	394	306	485	221	0	1761
Azithromycin	268	197	143	401	90	12	1111
Metronidazol	74	120	68	428	47	0	737
Roxithromycin	99	115	120	268	48	5	655

Table S11: Distribution of bacterial genera by treatment type.

Bacterium	Outpatient	Normal ward	Intensive care unit	Other treatment	Intermediate care / Awake station	Rehabilitation	Operating room	Unknown	Day clinic	Total
Escherichia	1946292 (41.2)	2337877 (49.4)	340394 (7.2)	59984 (1.3)	24352 (0.5)	9340 (0.2)	5859 (0.1)	2470 (0.1)	2299 (0.0)	4728867 (36.4)
Staphylococcus	959447 (44.9)	922832 (43.2)	199725 (9.3)	32897 (1.5)	15150 (0.7)	2549 (0.1)	1986 (0.1)	2209 (0.1)	1218 (0.1)	2138013 (16.5)
Klebsiella	746932 (47.1)	559248 (35.3)	236704 (14.9)	19994 (1.3)	13857 (0.9)	6062 (0.4)	1276 (0.1)	1043 (0.1)	857 (0.1)	1585973 (12.2)
Pseudomonas	476004 (48.8)	288481 (29.6)	181161 (18.6)	13513 (1.4)	8293 (0.9)	5256 (0.5)	508 (0.1)	770 (0.1)	476 (0.0)	974462 (7.5)
Proteus	453020 (48.8)	376870 (40.6)	74989 (8.1)	13841 (1.5)	5162 (0.6)	2506 (0.3)	719 (0.1)	818 (0.1)	490 (0.1)	928415 (7.1)
Streptococcus	165398 (25.1)	452040 (68.6)	29258 (4.4)	7609 (1.2)	1577 (0.2)	263 (0.0)	1903 (0.3)	353 (0.1)	196 (0.0)	658597 (5.1)
Enterobacter	248562 (50.5)	144078 (29.3)	85010 (17.3)	6746 (1.4)	4782 (1.0)	1727 (0.4)	356 (0.1)	539 (0.1)	288 (0.1)	492088 (3.8)

Continued on next page

Table S11 – continued from previous page

Bacterium	Outpatient	Normal ward	Intensive care unit	Other treatment	Intermediate care / Awake station	Rehabilitation	Operating room	Unknown	Day clinic	Total
Enterococcus	298863 (61.9)	36734 (7.6)	134153 (27.8)	4854 (1.0)	6320 (1.3)	1449 (0.3)	131 (0.0)	440 (0.1)	202 (0.0)	483146 (3.7)
Citrobacter	158982 (45.6)	140228 (40.2)	40230 (11.5)	4835 (1.4)	2440 (0.7)	1079 (0.3)	277 (0.1)	300 (0.1)	160 (0.0)	348531 (2.7)
Serratia	91632 (44.6)	66702 (32.4)	42170 (20.5)	2348 (1.1)	1612 (0.8)	780 (0.4)	126 (0.1)	185 (0.1)	100 (0.0)	205655 (1.6)
Morganella	83574 (50.0)	65850 (39.4)	13515 (8.1)	2434 (1.5)	833 (0.5)	332 (0.2)	175 (0.1)	201 (0.1)	89 (0.1)	167003 (1.3)
Haemophilus	26437 (19.8)	94005 (70.5)	11593 (8.7)	807 (0.6)	243 (0.2)	64 (0.0)	72 (0.1)	28 (0.0)	37 (0.0)	133286 (1.0)
Acinetobacter	38697 (44.9)	29615 (34.4)	14973 (17.4)	1422 (1.6)	759 (0.9)	542 (0.6)	89 (0.1)	57 (0.1)	33 (0.0)	86187 (0.7)
Providencia	21168 (49.9)	16989 (40.1)	3060 (7.2)	756 (1.8)	206 (0.5)	92 (0.2)	35 (0.1)	62 (0.1)	24 (0.1)	42392 (0.3)

Continued on next page

Table S11 – continued from previous page

Bacterium	Outpatient	Normal ward	Intensive care unit	Other treatment	Intermediate care / Awake station	Rehabilitation	Operating room	Unknown	Day clinic	Total
Mycobacterium	6022 (71.9)	1534 (18.3)	346 (4.1)	140 (1.7)	24 (0.3)	3 (0.0)	308 (3.7)	3 (0.0)	0 (0.0)	8380 (0.1)
Neisseria	387 (7.8)	4481 (90.6)	6 (0.1)	44 (0.9)	0 (0.0)	0 (0.0)	28 (0.6)	2 (0.0)	0 (0.0)	4948 (0.0)
Salmonella	1962 (41.9)	2445 (52.2)	209 (4.5)	36 (0.8)	15 (0.3)	3 (0.1)	9 (0.2)	1 (0.0)	0 (0.0)	4680 (0.0)
Shigella	9 (34.6)	16 (61.5)	1 (3.8)	0 (0.0)	0 (0.0)	0 (0.0)	0 (0.0)	0 (0.0)	0 (0.0)	26 (0.0)
Obesumbacterium	5 (62.5)	0 (0.0)	3 (37.5)	0 (0.0)	0 (0.0)	0 (0.0)	0 (0.0)	0 (0.0)	0 (0.0)	8 (0.0)

Table S12: Summary of clustering similarity metrics across evaluated configurations.

Metric	ICS	Stability	Hierarchical	N_{clusters}
Cosine	2.39	0.78	0.38	12.22
Dice	3.08	0.84	0.39	13.93
Jaccard	4.11	0.85	0.45	20.38
Phi	6.27	0.88	0.43	18.16

Table S13: Temporal summary of *Escherichia coli* blood and urine isolate cohorts (2019–2023).

Specimen	Year	Isolates	Features
blood	2019	40255	85
blood	2020	44435	86
blood	2021	44255	86
blood	2022	51407	86
blood	2023	58518	85
urine	2019	536878	87
urine	2020	616142	86
urine	2021	653464	87
urine	2022	705055	86
urine	2023	848709	86

Table S14: Cohort sizes for stratified comparisons across pathogens, specimens, and clinical contexts.

Pathogen genus	Specimen	Context dimension	Group 1	N_1	Group 2	N_2	N_{total}
Escherichia	Blood Culture	CareType	In-Patient	198538	Out-Patient	40279	238817
Escherichia	Blood Culture	OrgType	Hospital	238870	Doctor’s office	238870	477740
Escherichia	Blood Culture	ARS_WardType	Intensive Care Unit	35964	Normal Ward	154621	190585
Escherichia	Urine	CareType	In-Patient	1398165	Out-Patient	1957421	3355586
Escherichia	Urine	Care_Complexity	Tertiary & Specialized	789596	Primary/Secondary	807521	1597117
Escherichia	Urine	OrgType	Hospital	3360248	Doctor’s office	3360248	6720496
Escherichia	Urine	Sex	Woman	2324014	Man	627442	2951456
Escherichia	Urine	ARS_WardType	Intensive Care Unit	101328	Normal Ward	1238255	1339583
Klebsiella	Blood Culture	Care_Complexity	Tertiary & Specialized	39417	Primary/Secondary	34642	74059
Staphylococcus	Blood Culture	ARS_WardType	Intensive Care Unit	34401	Normal Ward	93355	127756

Table S15: Interactive network visualization files for *E. coli* co-testing analyses.

Analysis group	File description	Link
**A. Similarity Metrics (Co-Testing Patterns)**		
**Row 1** Jaccard	Blood culture co-testing network	explore
**Row 2** Dice	Blood culture co-testing network	explore
**Row 3** Cosine	Blood culture co-testing network	explore
**Row 4** Phi	Blood culture co-testing network	explore
**B. Temporal Trends (Use Case 1)**		
**Row 5** Blood 2019	Escherichia co-testing patterns	explore
**Row 6** Blood 2020	Escherichia co-testing patterns	explore
**Row 7** Blood 2021	Escherichia co-testing patterns	explore
**Row 8** Blood 2022	Escherichia co-testing patterns	explore
**Row 9** Blood 2023	Escherichia co-testing patterns	explore
**Row 10** Urine 2019	Escherichia co-testing patterns	explore
**Row 11** Urine 2020	Escherichia co-testing patterns	explore
**Row 12** Urine 2021	Escherichia co-testing patterns	explore
**Row 13** Urine 2022	Escherichia co-testing patterns	explore
**Row 14** Urine 2023	Escherichia co-testing patterns	explore
**C. Clinical Setting Divergence (Use Case 2)**		
**Row 15** Blood – Care (Community)	Community/Outpatient context	explore
**Row 16** Blood – Care (Inpatient)	Inpatient/Hospital context	explore
**Row 17** Blood – Ward (General)	General Ward context	explore
**Row 18** Blood – Ward (ICU)	ICU context	explore
**Row 19** Urine – Care (Community)	Community/Outpatient context	explore
**Row 20** Urine – Care (Inpatient)	Inpatient/Hospital context	explore
**Row 21** Urine – Ward (General)	General Ward context	explore
**Row 22** Urine – Ward (ICU)	ICU context	explore

5 Dataset Aggregation Rules

5.1 Overview

Primary analyses were conducted on the complete isolate-level dataset. Aggregation is performed exclusively for data sharing and replication. The aggregation pipeline is deterministic and identical for both the full dataset and the randomly sampled subset provided for verification.

5.2 Input Structure

Each row in the isolate-level dataset represents a single clinical isolate. Epidemiological covariates (e.g., Pathogen, CareType, Year) are categorical variables. Antibiotic testing indicators are binary variables with suffix `_Tested`, where

$$1 = \text{tested}, \quad 0 = \text{not tested}.$$

5.3 Grouping and Collapsing

Aggregation is performed within predefined strata defined by a selected subset of covariates. Let $\mathcal{G}$ denote isolates within one stratum and $N = |\mathcal{G}|$.

Selected covariates excluded from the grouping structure are intentionally collapsed to mitigate re-identification risk. Collapsed variables are encoded as "–", indicating marginalization rather than missingness.

5.4 Contingency Construction

Within each stratum, antibiotic indicators form a binary matrix

$$X \in \{0,1\}^{N \times M},$$

where M is the number of antibiotics.

For each unordered antibiotic pair (A_i, A_j), a 2×2 contingency table is computed:

	$A_j = 1$	$A_j = 0$
$A_i = 1$	a_{ij}	b_{ij}
$A_i = 0$	c_{ij}	d_{ij}

with

$$\begin{aligned} a_{ij} &= \sum_{k \in \mathcal{G}} X_{ki} X_{kj}, \\ b_{ij} &= \sum_{k \in \mathcal{G}} X_{ki} (1 - X_{kj}), \\ c_{ij} &= \sum_{k \in \mathcal{G}} (1 - X_{ki}) X_{kj}, \\ d_{ij} &= \sum_{k \in \mathcal{G}} (1 - X_{ki})(1 - X_{kj}), \end{aligned}$$

such that $N = a_{ij} + b_{ij} + c_{ij} + d_{ij}$.

5.5 Computation

Joint counts are computed via matrix multiplication:

$$A = X^{\top} X,$$

where $A_{ij} = a_{ij}$. Let $s_i = \sum_{k=1}^{N} X_{ki}$. Then

$$b_{ij} = s_i - a_{ij}, \quad c_{ij} = s_j - a_{ij}, \quad d_{ij} = N - a_{ij} - b_{ij} - c_{ij}.$$

This vectorised formulation ensures computational efficiency and exact reproducibility.

5.6 Output Structure

For each stratum and antibiotic pair, the exported dataset contains grouping covariates, collapsed covariates encoded as "–", antibiotic identifiers (`ab_1`, `ab_2`), and counts $(a_{ij}, b_{ij}, c_{ij}, d_{ij})$.

Table S16 shows an excerpt of the aggregated output for *Escherichia coli* blood culture isolates in 2019.

Table S16: Example aggregated contingency output for one stratum.

PathogenGenus	CareType	Year	...	ab_1	ab_2	a	b	c	d
Escherichia	In-Patient	2020	...	CEZ - Cefazolin	CMP - Chloramphenicol	52	197	5607	34387
Escherichia	In-Patient	–	...	CEZ - Cefazolin	DOX - Doxycyclin	3260	466	2399	34118
Escherichia	In-Patient	2020	...	CEZ - Cefazolin	FLU - Flucloxacillin	0	0	5659	34584
Escherichia	In-Patient	2019	...	CEZ - Cefazolin	GEN - Gentamicin	5622	34064	37	520
Escherichia	In-Patient	–	...	CEZ - Cefazolin	MCL - Mecillinam	0	706	5659	33878
Escherichia	In-Patient	2022	...	CEZ - Cefazolin	MTR - Metronidazol	0	1	5659	34583
Escherichia	Out-Patient	2023	...	CEZ - Cefazolin	NFT - Nitrofurantoin	2535	6073	3124	28511
Escherichia	Out-Patient	2020	...	CEZ - Cefazolin	OXA - Oxacillin	55	292	5604	34292
Escherichia	Out-Patient	–	...	CEZ - Cefazolin	SXT - Co-Trimoxazol	5657	34320	2	264
Escherichia	–	2020	...	CEZ - Cefazolin	TET - Tetracyclin	2300	2777	3359	31807
Escherichia	In-Patient	2021	...	CEZ - Cefazolin	TRP - Trimethoprim	2194	1384	3465	33200

Only aggregated counts are exported. No isolate-level records or uniquely identifying combinations of metadata are retained.
